# Technology-Based Tools for English Literacy Intervention: Examining Intervention Grain Size and Individual Differences

**DOI:** 10.3389/fpsyg.2019.02625

**Published:** 2019-11-26

**Authors:** Beth A. O’Brien, Malikka Habib, Luca Onnis

**Affiliations:** ^1^National Institute of Education, Nanyang Technological University, Singapore, Singapore; ^2^Department of Education Sciences, University of Genoa, Genoa, Italy

**Keywords:** struggling readers, technology, intervention, statistical learning, phonological awareness

## Abstract

Technology plays an increasingly important role in educational practice, including interventions for struggling learners (Torgesen et al., 2010; de Souza et al., 2018). This study focuses on the efficacy of tablet-based applications (see Word Reading, Grapholearn, and an experimental word-level program) for the purpose of supplementing early English literacy intervention with primary grades 1 and 2 children. The children were identified for learning support programs within Singaporean schools, which follow a bilingual policy, meaning children were learning reading in English plus an additional language. One hundred forty-seven children across seven schools participated (Mean age = 6.66). Within learning support classrooms, triplets of students matched on basic reading skills were randomly assigned to one of three groups: (1) phoneme-level, (2) rime-level, or (3) word-level focused interventions. All groups performed reading skills activities on iPads, across two phases over a 14-week period. Assessments for word reading accuracy and fluency, pseudoword decoding accuracy and fluency, and spelling were administered at four time points, pre- and post-intervention. Additional baseline measures were taken to assess individual differences in phonological awareness, orthographic awareness, general cognitive ability, statistical learning, and bilingual vocabulary knowledge. Mixed model analysis was conducted on the pre- to post-test measures across the two phases of the intervention (focused on accuracy then fluency). All groups made gains across the different literacy measures, while the phoneme-level intervention showed an advantage over the rime-level intervention, but not the word-level intervention, for decoding. There were also moderating effects of individual differences on outcomes. The general pattern of results showed an advantage of the word-level intervention for those with poorer phonological awareness for reading fluency; and a phoneme-level intervention advantage for those with poorer statistical learning ability. Children’s bilingual group (English plus Mandarin, English plus Malay, or English plus Tamil) also showed differential effects of the type of intervention (e.g., phoneme- or word-level) on different outcome measures. These results, along with data collected from the tablets during the intervention, suggest the need to examine the interplay between different types of technology-based interventions and individual differences in learning profiles.

## Introduction

Since the first evident writing system in 1800 BCE (Wolf and O’Brien, 2006), several iterations of invented symbolic representations of language emerged and have persisted to the present day – the prolific alphabetic systems, along with alphasyllabaries and morphosyllabaries. The glacial-speed changes to these invented writing systems seem to have met with an evolutionary leap currently upon us – the technology-supported renditions of script. New possibilities of interacting with script that is responsive and dynamic creates different environments for processing text as a reader. At the same time, new environments are made possible for learning to read. It is important to consider how reading occurs on a cognitive level, and by extension how reading is learned, as half of the equation in the human–machine interface of reading on modern electronic digital devices. The focus of the current study is on teaching children to read in English with the use of technology-mediated applications. In particular, we center on children who are struggling learners, and in this case also bilingual learners who are learning to read in an additional language along with English.

Technology-based environments for instruction and intervention have some advantages over traditional methods, in that they are engaging, reduce social pressure to perform, are adaptable to individual performance with features like embedded scaffolding and feedback, as well as the crucial ingredient for struggling learners – extensive practice (Clark et al., 2016; Laurillard, 2016; de Souza et al., 2018). Nevertheless, meta-analytic findings report better student progress with teacher-based versus computer-based interventions (Dowker, 2005; Slavin et al., 2011), but these findings do not account for differences across computer-based programs, where some approaches may be more beneficial than others. Rather than being considered as a replacement for human-led instruction, it is suggested that technology-based approaches serve primarily as tools that can be used to remediate or optimize learning experiences for all individuals (Dowker, 2005; Rose and Strangman, 2007). Accordingly, it is recommended that technology-based instruction conforms to known learning and pedagogical principles (e.g., Butterworth and Yeo, 2004; Hirsh-Pasek et al., 2015); that is by “using the combination of images and sounds and through a paradigm that tries to understand human behavior and, as well, employ an approach that matches how effective teaching actually occurs” (de Souza et al., 2018, p. 7).

An unresolved debate concerns what is the most effective teaching approach for English literacy acquisition. This includes questions about the optimal input for learning to read in the English language (National Reading Panel (US) et al., 2000; Walton et al., 2001; Hatcher et al., 2006), especially for struggling learners and children learning to read in multiple languages, as in the present study (Rickard Liow and Lau, 2006). Should instruction be aimed at coding of the individual phoneme, or sublexical rime patterns, or even whole words? This question is especially relevant to reading in English, because English is not considered as an ‘ideal’ alphabetic system with clear mappings of letters to speech sounds (Caravolas, 2004). English has a deep orthography, beyond simple 1:1 mappings to the phonology, and it is even described as more of a morphophonemic system than a strictly alphabetic one (Nagy et al., 2006). The deep orthography means that sometimes letters are pronounced different ways, or sounds are spelled differently; yet there is solace in larger contextual units in terms of spelling-sound consistency. Rime patterns are more consistent than individual vowels, and the preceding consonant can provide information about how a vowel should be pronounced (Treiman et al., 2002, 2006). As a ‘non-ideal’ alphabetic system, English presents a challenge to beginning readers (Seymour et al., 2003).

Beginning readers have to learn the mapping system between phonology and orthography (Perfetti and Veroeven, 2017). To understand this mapping system, they need to be able to identify phonological units within words in order to map them to corresponding orthographic symbols (e.g., letters). Knowledge about the language’s orthography as well as an awareness of the phonology are thus two requisites for learning this mapping system. Orthographic awareness involves knowledge about the structure of written language, in terms of where letters tend to appear within words and permissible letter sequences, while phonological awareness involves the ability to identify, segment, and manipulate speech sounds within words. Ample evidence supports the close and predictive role of phonological awareness to reading ability across alphabetic writing systems (Lonigan et al., 2000; Melby-Lervåg and Lervåg, 2011; Branum-Martin et al., 2012). Thus, phonological awareness is held as a central mechanism for learning to read alphabetic languages.

Additionally, or alternatively, it is suggested that reading may be mediated by statistical learning mechanisms (Seidenberg and McClelland, 1989). Statistical learning involves the ability to pick up probabilistic properties of information, usually implicitly. It is argued that the process of learning to read involves implicitly picking up the mapping system of speech and print (phonology – orthography) as a set of statistical regularities (Steacy et al., 2017; Sawi and Rueckl, 2018). Steacy et al. (2017) propose an individual differences model in which statistical learning is a key mechanism that impacts how children are able to avail of learning opportunities in their environment – or not, in the case of struggling learners. In support of this, performance on statistical learning tasks correlates with reading ability across a range of ages (Arciuli and Simpson, 2011).

Struggling readers, or children with developmental dyslexia, show an array of anomalies in terms of performance on measures of phonological awareness, rapid symbol naming, orthographic awareness (Norton and Wolf, 2012; Peterson and Pennington, 2012; Wandell et al., 2012), and, according to more recent findings, statistical learning (Aravena et al., 2013; Gabay et al., 2015; Sawi and Rueckl, 2018). Measures of phonological awareness robustly discriminate typical from atypical readers, including findings across adults, children and at-risk pre-readers (Pugh et al., 2000; Hampson et al., 2004; Hoeft et al., 2006; Saygin et al., 2013). Moreover, reduced performance on phonological awareness tasks correlates with neurophysiological anomalies in regions of the reading circuit of the brain (Saygin et al., 2013), and reported deficits in phonological awareness persist across development for individuals with dyslexia (Goldstein and Kennemer, 2005). Statistical learning ability predicts reading ability within groups with dyslexia (Gabay et al., 2015), and dyslexic individuals show poorer performance on implicit learning tasks related to sounds and letter-to-sound matching (Aravena et al., 2013; Gabay et al., 2015). Findings support the role of statistical learning in dyslexia, although this may not be as consistent as those with phonological awareness (Sawi and Rueckl, 2018). Thus, these possible mechanisms for learning to read, phonological awareness and statistical learning, may affect student learning, and so we focus on these as possible moderators of intervention effects for struggling learners.

Furthermore, biliterate bilinguals demonstrate cross-linguistic and cross-orthographic influence (Koda, 2005; Geva, 2014; Lallier and Carreiras, 2018), raising questions about approaches to training. Different writing systems vary in their cognitive demands, including levels of metalinguistic awareness. Knowledge about phonology is evidently important for learning to read English and other alphabetic languages, but some languages are more easily decodable at the phoneme level (e.g., Italian, Tamil), versus the syllable level (e.g., English, Malay), while others are morphemically more transparent (e.g., Chinese) (Bassetti, 2013). The unit level or grain size of reading for bilingual readers is hypothesized as a hybrid of the optimal grain-sizes per their known languages (Lallier and Carreiras, 2018). Thus, of concern is whether promoting awareness at the phoneme level has positive effects across languages, or may be simply confusing for some bilingual children who learn to spell in one language at the syllable or morpheme level (e.g., Rickard Liow and Lau, 2006, p. 876). Therefore, we also considered in our analysis the other language that the children were learning in school, simultaneous with English, and how this played out with intervention effects.

Thus, in the current study on technology-mediated reading intervention, we consider the debated optimal input for learning to read English – at the level of either the phoneme, rime or whole word unit. Previous studies found that computer assisted reading training with speech-feedback was most beneficial when feedback was directed at either syllable or onset-rime units as compared with whole words (Olson and Wise, 1992; Ecalle et al., 2009), while a study with graphics-based feedback showed a trend for better benefits with a focus at the rime-level versus the phoneme level (Kyle et al., 2013). However, given the range and heterogeneity of difficulties that individual struggling learners show, it is also quite possible that certain types of intervention are more beneficial for different types of learners than others (e.g., see Cheung and Slavin, 2013). Therefore, we consider the question of the optimal input unit-size along with individual differences that may moderate such effects.

In the current study with early primary school (grades 1–2) children learning to read in English within Singapore, we address the following research questions:

(1) What is the optimal grain size for teaching struggling learners the phoneme–grapheme correspondences of English? We investigate this question using a randomized controlled design with three intervention groups, focusing technology-mediated intervention at (a) the phoneme-level, (b) rime-level, or (c) the word-level. Two phases of instruction focus on, first, explicit teaching and learning of GPC through iPad-based activities for developing accuracy for phoneme–grapheme correspondence (GPC). The second phase extends the learning of GPC accuracy to fluency through iPad-based activities that require rapid matching of orthographic patterns to an auditory stimulus. We hypothesize that the word level group would show least progress, as lexical processing would be less efficient than to learn sublexical GPC patterns.
(2) Do individual characteristics of struggling learners moderate the effect of intervention? We include baseline measures of individual performance on phonological awareness and statistical learning, along with orthographic awareness and rapid naming measures to examine possible interactions with learning outcomes. Also, while English is the language of instruction in Singapore, children are exposed to and are taught early literacy skills in their additional language (Mandarin or Malay or Tamil). Therefore, we also consider individual differences in line with the sets of scripts that each child is learning in school. We hypothesize that phonological awareness may be more relevant for the phoneme level intervention, since lexical strategies could be used for the word level intervention activities, such that phonological awareness would positively moderate outcomes for the phoneme-level group. Also, we hypothesize that statistical learning may be most beneficial with the rime level intervention, because picking up orthographic patterns would be easier for those with greater statistical learning ability. Therefore, we would predict that phonological awareness moderates outcomes for intervention focused at the phoneme level, and statistical learning moderates outcomes for intervention focused at the rime level.

## Materials and Methods

### Participants

One hundred forty-eight children from seven primary schools in geographically dispersed locations across Singapore participated (Mean age = 79.91 months, *SD* = 4.82, at the beginning of the study). One hundred and thirty-six were entering primary grade 1, and 12 primary grade 2. The children were identified as at risk for reading difficulties, and were enrolled into learning support programs (LSPs) within Singaporean schools. Informed consent was obtained for all participants from a parent, along with child assent, in accordance with the Declaration of Helsinki. Procedures were approved by and followed ethical standards of the research team’s university institutional review board. Within learning support classrooms, triplets of students matched on basic reading skills (British Ability Scales-III) were randomly assigned to one of three intervention groups: (a) phoneme-level, (b) rime-level, or (c) word-level focused interventions. Within each intervention group, there were 58 English–Chinese, 73 English–Malay, and 17 English–Tamil speakers, where the composition of bilingual groups did not differ across intervention conditions [X ^2^(4) = 2.57, *p* > 0.05]. Further, the intervention groups did not differ in age [*F*(2,145) = 0.078, *p* > 0.05], nor on baseline measures of cognitive ability and vocabulary (refer to Table 1).

**TABLE 1:** Baseline measures across intervention groups.

	**Phoneme**		**Rime**		**Word**		
**Baseline measure**	***M***	***SD***	***M***	***SD***	***M***	***SD***	***F, p***
Reading achievement	7.64	10.08	6.09	6.51	6.93	8.49	0.169, 0.85
Non-verbal ability	86.62	8.00	87.58	7.83	86.12	7.46	0.373, 0.69
Memory for digits	6.56	5.68	7.31	5.73	7.23	4.95	0.354, 0.70
Vocabulary English	26.85	11.98	22.76	12.92	22.92	12.59	1.416, 0.25
Vocabulary other	35.86	12.45	31.93	12.15	32.54	11.49	0.798, 0.45
Phonological awareness	7.17	5.35	6.92	4.55	7.75	5.96	0.369, 0.69
Rapid symbol naming	40.13	22.28	40.53	17.47	42.06	48.07	0.037, 0.96
Orthographic awareness	22.69	4.05	21.40	4.57	21.30	5.05	0.301, 0.74

*Reading achievement and non-verbal ability was assessed with BAS-3 (Elliott and Smith, 2011) and are reported as total correct raw scores; Vocabulary for English and the child’s other language (Mandarin, Malay, or Tamil) was assessed with the BLAB (Rickard Liow and Sze, 2008); Memory for digits, phonological awareness, and rapid symbol naming were assessed with the CTOPP-2 (Wagner et al., 2013); Orthographic awareness measure was adapted (Cunningham et al., 2001; O’Brien, 2014). For each measure, total correct raw scores are reported, except for rapid symbol naming which is reported in total seconds taken to complete all items.*

### Measures

Baseline measures were taken prior to pretest. Outcome measures of reading and decoding accuracy and fluency, plus spelling were taken at four time points: pre-test prior to intervention, mid-test at the end of intervention phase one, post-test at the end of phase two of the intervention, and follow-up 3 months after post-test.

#### Baseline Measures

*General Cognitive Ability* was assessed with the British Ability Scales III Quantitative Reasoning (SET B; Rasch split-half reliability = 0.87–0.90) and Matrices (reliability = 0.83–0.87) subtests (Elliott and Smith, 2011). Children view sets of number pairs and are asked to find the relationship between the pairs in order to complete additional number sets (by filing in blanks with the corresponding number). Administration was discontinued after three consecutive errors. In the Matrices subtest, children view a matrix of 9 figures including one blank, and they have to choose a figure, from 4 to 6 options, to complete the matrix pattern. Administration was discontinued after three consecutive errors. An overall summed score from both tests was used as an indicator of cognitive ability and was entered as a covariate. Raw scores were used because local norms are not currently available for this test.

*Verbal memory* was assessed using the Memory for Digits subtest of the Comprehensive Test of Phonological Processing (CTOPP-2; Wagner et al., 2013). Children were given a series of digits and asked to repeat them backward. This subtest is made up of 21 items. A score of 1 was given for correct response. The task was discontinued when the child made three consecutive incorrect responses.

*Receptive Vocabulary* was assessed using the Bilingual Language Assessment Battery (BLAB; Rickard Liow and Sze, 2008; split-half reliability for English = 0.85 and for Mother Tongue = 0.80, from a local sample). The BLAB is a locally developed measure that has been used in multiple published studies in Singapore. Both vocabulary tests follow the same format, where, on each trial, children listened to an audio-recorded word and selected one of four pictures on the iPad screen that matched the word. Children completed this task in both the English language and the child’s Mother Tongue or heritage language (Mandarin, Malay, or Tamil) that they were also learning in school. In both English and Mother Tongue versions of the BLAB, children first completed three practice trials with corrective feedback, followed by 80 experimental trials. The final score for each child was the total number of correct responses on the experimental trials.

*Basic reading skill* was assessed at baseline with the British Achievement Scale III Reading subtest (Elliott and Smith, 2011), using form Word Reading form A (Rasch split-half reliability = 0.99). The task was administered according to the guidelines, whereby all children started with item 1 and were asked to read aloud a series of words presented on a stimulus card. Testing was discontinued when the child made 8 errors in a block of 10 words. Items were scored according to locally accepted standards of pronunciation, with 1 point awarded per correct response. Total number of correct words was summed for the final score.

*Phonological awareness* was assessed in English using the Comprehensive Test of Phonological Processing Elision subtest (CTOPP-2; Wagner et al., 2013, split-half reliability = 0.95 from a local sample). Children were required to listen to a word (e.g., toothbrush), repeat it, and then say what is left of that word after dropping designated word (e.g., brush) or sound segments (e.g., cup without the sound/k/). Corrective feedback was given on the first 10 items. Test administration was discontinued after three consecutive errors. An overall total correct score was used as an indicator of phonological awareness.

*Rapid symbol naming* was assessed in English with the CTOPP-2 RAN letters subtest (Wagner et al., 2013) (test–retest reliability = 0.90). In this test, the children named sets of letters that were presented in 4 rows by 8 columns. Time to complete naming all items was scored in seconds.

*Orthographic awareness* was measured using an orthographic choice task (Cunningham et al., 2001), and a wordlikeness judgment task (Cunningham et al., 2001; O’Brien, 2014). For the orthographic choice task, children had to distinguish words from non-word letter strings that could be pronounced identically (e.g., rain–rane). The task included 23 trials. For the wordlikeness task, children decided which of two letter-strings looked more like a real word (e.g., beff-ffeb). This task included 19 trials. Stimuli for each task were presented on an iPad, and children completed all items (split-half reliabilities = 0.51–0.84, Cunningham et al., 2001). The total correct score was summed for the two tasks.

*Statistical Learning* (SL) was assessed using a visual SL test similar to Arciuli and Simpson (2011) and Raviv and Arnon (2018). The SL test comprised two phases: (1) a training phase, followed by (2) a surprise forced-choice test phase (refer to Appendix). Four base triplets of 12 cartoon figures described as “aliens” were chosen as stimuli for the task. *Training Phase 1.* The triplets were presented as a continuous stream of aliens queuing up to board a space-ship. Aliens were shown one at a time, in the center of the iPad display against a black background (each visible for 500 ms with an interstimulus interval of 100 ms). Each triplet appeared 24 times. There were also 24 occasions where a repeated presentation of one alien was given, and the child’s task was to detect these instances and press a button. *Test Phase 2.* After completing phase 1, children were given a ‘test’ task, for which they were asked to identify alien triplets that had appeared together previously. In a 2-AFC they chose whether the triplet on the left or right of the screen had appeared together before. Overall, the average response rate was 50% correct (with a *SD* = 10.2%). This mean is low compared with similar aged typically developing children in Raviv and Arnon (2018), who reported a 52% response rate by 5 to 6 year olds, and 57% by 6 to 7 year olds on a similar task. The poorer performance may not be unexpected given that the current sample includes struggling readers, who have been shown to have poorer statistical learning in some studies (Aravena et al., 2013; Gabay et al., 2015). Scores were calculated as the difference between the raw score and chance level (16), with raw scores below 16 recast as 0’s.

#### Outcome Measures

*Reading and decoding accuracy* was assessed four times with the letter-word identification and word attack subtests of the Woodcock-Johnson III Tests of Achievement (WJIII; Woodcock et al., 2007; test–retest reliability = 0.87 and 0.91). Children had to identify letters first and then pronounce words or decode pronounceable non-words, or pseudowords. The test was discontinued when the child had six incorrect responses. The total number of correctly read words or pseudowords was taken as the final score. Accuracy scores for word reading and decoding were converted to grade equivalent scores based on the published norms of the WJIII (Woodcock et al., 2007), given that there may be age differences between the US-based normative sample and the current Singaporean sample for children in primary grades 1 and 2.

*Reading and decoding fluency* was assessed four times with the sight word efficiency and phonemic decoding efficiency subtests of the Test of Word Reading Efficiency (TOWRE-2; Torgesen et al., 2012; test–retest reliability = 0.97, 0.96, respectively, according to a local normative sample). A practice test was first given to obtain confirmation that the child understood the directions. The task was to read aloud as many words, then pseudowords from separate lists as quickly as possible within 45 s for each list. The total number of correctly read words or pseudowords was taken as the final score. Normative data from a local sample of Singaporean primary school children were used to calculate z-scores for these reading and decoding tasks (Chen et al., 2016).

*Spelling* was assessed four times with the British Achievement Scale III Spelling subtest (Elliott and Smith, 2011; Rasch split-half reliability = 0.96–0.97). The spelling subtest was administered following the guideline whereby all children started with the first item, and they completed all items (10) in the first block. Thereafter, administration was conducted item-by-item until they committed 8 or more errors starting from the second block. Research assistants introduced the spelling task by pointing to the spelling worksheet corresponding to the child’s starting item. Then the research assistant read the target word, read the sentence with the target word, and then repeated the target word (e.g., *on*. I lie *on* the grass. *on*). The total number of correctly spelled words was taken as the final score.

### Procedures

#### Assessments

Child-based assessments were administered at four time points: at the beginning of the academic year, prior to intervention (baseline and pre-test in February) before the mid-year school break, between phases 1 and 2 of intervention (mid-test in May); at the end of the academic year, after both phases of the intervention (post-test in October); and at the start of the following academic year, 3 months after the intervention (follow-up in February the following year). The battery of tasks was given in two to three sessions for each time-point, with each set taking 30 to 60 min to complete. Tests were given in the same order, and each task was administered individually to the child.

#### Experimental Conditions

There were 46 students per intervention group across schools. Within each classroom, the randomly-assigned matched sets of participants took part in one of three interventions: phoneme-, rime-, and word-level intervention, across two 7-week phases of intervention. The first intervention phase was focused on training accuracy of GPCs (with the *SeeWord Reading* app) for the phoneme and rime groups, and a word-level reading app for the word group. The second intervention phase was focused on training fluency of GPCs (with the *Grapholearn* app) for the phoneme and rime groups, while the word-level group continued with a word- and sentence-level app designed for this study. The phoneme group received the *Grapholearn-Phoneme* (GLP) app, and the rime group the *Grapholearn-Rime* app (GLR) to contrast conditions based on two opposing theoretical views of how phonics should be taught: either at the small unit size (e.g., following synthetic phonics, Hulme et al., 2002), or at the rime level (e.g., following learning with analogies, Goswami and Bryant, 1990). The word group served as a comparison between lexical level compared with the sublexical level focus of intervention (see Table 2).

**TABLE 2:** Intervention activities per group.

**Experimental group**	**Intervention phase 1 (7 weeks)**	**Intervention phase 2 (7 weeks)**
Phoneme level	*SeeWord Reading* (to learn GPC accuracy)	*Grapholearn* – Phoneme (to develop GPC fluency)
Rime level	*SeeWord Reading* (to learn GPC accuracy)	*Grapholearn* – Rime (to develop GPC fluency)
Word level	Activities to match words with pictures (for meaning), to select their correct spelling, and to read words aloud.	Activities to match sentences with pictures (for correct meaning), and to read sentences aloud

*GPC = grapheme-to-phoneme correspondence; App = iPad tablet-based applications.*

#### Instruction

Each school’s LSP coordinators or teachers were trained to administer the intervention to small groups. Each child within the group worked independently on their own iPad. LSP staff provided instruction on how to use the apps by demonstrating the procedures of the app, and they explained the purpose so that the child understood the point of the lesson. If a child had any difficulty interacting with the content presented in the app, the staff helped the child by explaining the content using simpler language. Instructions at each level were also given through the app and children used headphones to listen. The staff checked that the children understood how to use the app effectively before he or she worked on it independently. The trained LSP staff supervised children in all groups working individually with the iPad app for 10 min each day, 5 days per week during two 7-week phases of instruction (for a total of 28 lessons). The level of treatment intensity was in line with other similar intervention studies (e.g., Leafstedt et al., 2004; de Graaff et al., 2009; Yeung et al., 2012).

### Intervention

#### Phase 1 – Training Grapheme–Phoneme Accuracy

Both the phoneme- and rime-level groups were first trained with the *SeeWord Reading* app in a series of lessons that progressively teach students grapheme–phoneme correspondences for up to 44 speech sounds (or phonemes) in English. The *Seeword Reading* app is a digital, interactive tool that uses visual communication principles with picture-embedded fonts to provide graphic cues so students may concretely visualize the relationship of phonemic sounds to alphabetic letterforms (Seward et al., 2014). This intervention has been used successfully with small groups of kindergarteners in the US and Singapore (O’Brien and Chern, 2015).

In the app, letterforms are presented with pictures of objects that begin with the sound represented by the letter (e.g., a peapod embedded within the letter ‘p’). There are three levels in *SeeWord Reading* and each level records children’s audio and kinesthetic interactions: Letter-Sound Correspondence where three letters/sounds were taught each lesson in isolation (level 1), within words at the Word Building level (level 2) and within a connected story at the Story level (level 3). Three to four letters are presented in each lesson, and the order of presentation was based on the frequency of letters in English language (Common Core State Standards for English Language Arts and Literacy), wherein the most common consonant and vowel sounds were taught first in the program.

At the Letter-Sound Correspondence level, children were presented with one letter at a time and matching visual cues to help them to remember the phoneme–grapheme correspondences. For example, children were introduced to the letter ‘a’ and had to trace the letterform with their finger in the same way they had been taught to write the letter in class. Multiple attempts are allowed until the child draws in the most accurate direction possible. Thereafter, a sequence of images embedded in the letterform appears (e.g., alligator, apple etc.) as a visual cue for the child. Finally, the child had to name the letter, find the corresponding sound and a word that began with the sound.

At the next level, Word Building, children had to make words using the sounds they had learned in the previous Letter-Sound Correspondence level. For instance, after naming the letter ‘p’ and learning the corresponding sound/p/, a word rhyme pattern/an/may be presented with a blank at the beginning. Children were instructed to find the missing letter by choosing and pulling the letter tiles from the bottom of the screen to the word. Visual cues of the embedded pictures were presented in the letters when the child tapped on the letter, if they were unsure of its sound. Upon building a new word, children were instructed to read the word aloud and their voices were recorded for them to play back and listen to or re-record.

Within the final Story level, children listened to a story from the app and while the text was highlighted as the words were read. Subsequently, they were asked to locate and touch each letter in the story text that matched a given speech sound (phoneme). Positive reinforcement was provided in the form of stars when the correct letter was touched.

#### Training Word Identification and Reading

The word-level group worked on developing their reading skills with a series of iPad-based activities focused at the word level. The children worked with an in-house developed app, which was used to reinforce learning through vocabulary building and word reading games. The activities were aligned with the type of review activities that were typically conducted at the end of LSP lessons. There are five levels to the word reading game: (1) Picture Match, (2) Word Match, (3) Multiple Match, (4) Spelling Match, and (5) Flashcards. Each day of the week they practiced a different level, and each activity included feedback, while the child could only move on to the next trial after selecting the correct option.

Within the first level, Picture Match, children had to select the picture that corresponded to the word presented on the iPad. For instance, when presented with the word “sat,” the child selected the image that matched the word. If a wrong image was selected, for example a picture representing “tip” instead of “sat,” the picture would be highlighted in red and the word for that image was given as feedback. If the correct image for the word “sat” was selected, it was highlighted in green and the correct word was read out to the child. The child could also click on the sound icon on the screen to hear the word as many times as needed.

The next level, Word Match, included matching picture to the printed word shown on the iPad. For example, the word “tap” would appear on the screen and the child had to select one out of the three pictures corresponding to the meaning of “tap.” Similar feedback was given for an incorrect response (red highlighing with corrective feedback) and a correct response (green highlighting and the word being read) as in the previous level. Moving onto the Multiple Match level, three images and three words were shown on the screen and the children could click on the image to hear the corresponding word for each image. The child then had to drag each word under the correct image. If any of the words were matched to the wrong image, a buzzer rang and the word was dropped back down, for the child to retry.

In the fourth level, Spelling Match, one image was shown with three words (e.g., tan, tap, tin). The children dragged the correctly-spelled word to match the image. They could also click on the image to hear the word. Similar to the previous level, if an incorrect word was matched to the image, a buzzer sounded, and the word was dropped back to the original position. If the correct word was matched to the image, the child could proceed to the next question. In the last level, Flashcards, children read words presented on the iPad in a card deck with feedback. Their voice was recorded, then they could play their recording back and then listen to the correct recording of the word to check if they were accurate.

The research team worked closely with the LSP coordinators to develop the app to support the curriculum content, while at the same time ensuring that the activities were focused on lexical processing and were dissimilar from the sub-lexical focus of the other groups’ content. The children worked with the app on iPads individually for 10 min per day 5 days per week over the same 7 week period as the other groups.

#### Phase 2 – Training Grapheme–Phoneme Fluency

For the phoneme and rime groups, the second phase of the study involved playing *Grapholearn*, a computerized learning environment for learning to pair audio segments (phonemes, syllables, and words) with visual symbols (graphemes, words, etc.) in a timed format to encourage automatization (Richardson and Lyytinen, 2014). Feedback, positive and corrective, was provided and the game was adaptive to the player’s performance. The intervention has been widely used across multiple countries (e.g., Saine et al., 2011; Kyle et al., 2013).

The phoneme-level group played the *Grapholearn-Phoneme* version, where letter-sound correspondences were learned starting with the most frequent, most consistent, and most prototypical first and these were also reinforced first during later game streams. In streams 1 and 2, children were introduced to all the single letter-sound correspondences in English (e.g., ‘I,’ ‘a,’ ‘ee,’ ‘oa’). Following, in Stream 3, children were presented with phonemes that were blended into consonant-vowel (CV) units (e.g., /ti/, /loa/). Phonemes were combined into vowel-consonant (VC) units in Stream 4 and children had to combine the letter-sounds into larger units and finally create real words. Starting from Stream 5, children were presented with whole words and had to select letter-sound correspondences within the whole words or combine letter-sounds correspondences into whole words (Kyle et al., 2013).

The rime-level group played the *Grapholearn*-*Rime* version focusing on orthographic rime units. In each stream, children familiarize themselves with a single letter-sound correspondence, learned to combine the letter-sound into an orthographic rime unit with an onset and a rime pattern and finally into consonant-vowel-consonant (CVC) words. They then played games with matching rhyming words (Kyle et al., 2013). Children were also shown how the same letter-sounds may be broken down in terms of how the letters represent the constituent phonemes (e.g., “p + ad = pad,” then “pad = p-a-d”). The first stream included a small set of letter-sound correspondences (e.g., C, S, A, T, P, I, N). Rime units that were also real words were presented first (e.g., ‘at’ and ‘in’) and reinforced. For example, children had to blend the orthographic rime units (e.g., A, T, I, and N) together into units (‘at’ and ‘in’), then with an added onset sound to build words (like ‘cat’ and ‘tin’). Thereafter, rime units that were not real words were also introduced (e.g., ‘og’ and ‘ag’), allowing the creation of real words, such as dog and bag (Kyle et al., 2013). For both *GraphoLearn* apps, there were periodic mini assessments, where knowledge of symbol (grapheme) to sound matching was tested without feedback.

#### Training Word Identification and Reading

The word-level group completed iPad-based activities on the in-house developed app, to continue reinforcing word vocabulary, spelling, as well as sentence comprehension. The activities for the second phase of intervention included (1) Word Match, (2) Multiple Match, (3) Spelling Match and (4) Flashcards, and, by the end of phase, sentence-based activities for (5) Sentence Picture Match and (6) Sentence Build. The first four activities were similar to phase 1, but with different words. In the Sentence Picture Match, children see a picture and three short sentences (e.g., ‘They walk to the park’), then they picked which sentence matched the meaning of the picture. For Sentence Build, students had to listen to a sentence that was read out on the iPad. Thereafter, they selected words from a word bank at the bottom of the screen and dragged them into the appropriate place in the sentence. The children worked with the app on iPads individually for 10 min five times per day over the same 7 week period as the other groups. Children also read sentences for the Flashcards activity. For this, the child clicked on “rec” button to read the sentence into the microphone and clicked on the “play” button to listen to their own recordings, then they could check the correct pronunciation of the word using a “check” button.

## Results

For the reading outcome measures, raw scores for accuracy on word reading and decoding were converted to grade equivalent scores based on the published norms of the WJIII (Woodcock et al., 2007). Raw scores for fluency on word reading and decoding were converted to z-scores using a local normative sample of TOWRE scores (Chen et al., 2016). Spelling raw scores were scaled and centered within the current sample. Descriptive statistics for these measures at pretest are presented in Table 3.

**TABLE 3:** Reading and spelling pretest scores across intervention groups.

	**Phoneme**		**Rime**		**Word**	
**Measure**	***M***	***SD***	***M***	***SD***	***M***	***SD***
Word reading accuracy	2.05	(1.05)	1.90	(0.08)	2.05	(0.82)
Non-word reading accuracy	3.78	(3.82)	2.75	(0.19)	3.33	(2.06)
Word reading fluency	–1.11	(1.08)	–1.41	(0.12)	–1.01	(1.09)
Non-word reading fluency	–0.39	(0.90)	–0.64	(0.08)	–0.34	(0.82)
Spelling	14.61	(1.47)	13.29	(1.18)	15.31	(8.59)

*Word and Non-word Reading Accuracy = grade equivalent scores from the WJIII word identification and word attack subtests (Woodcock et al., 2007). Word and Non-word Reading Fluency = z-scores based on local sample from the TOWRE-2 sight word and phonemic decoding subtests (Torgesen et al., 2012; Chen et al., 2016). Spelling = raw scores from the BAS-3 (Elliott and Smith, 2011).*

Zero-order correlations were first run with all of the outcome measures at each time point and with the baseline measures of non-verbal cognitive ability, phonological awareness, statistical learning, rapid naming, and orthographic awareness, along with age. Table 4 shows the Pearson correlation coefficients for this full set of measures, between the dependent variables for reading and spelling at each time point and the other baseline measures. As shown, there are high correlations between the outcome measures of reading and spelling over time, and baseline measures also showed low to moderate correlations with outcome measures at most time points.

**TABLE 4:** Pearson correlations for reading and spelling outcome measures over time and baseline measures of individual differences.

	**1**	**2**	**3**	**4**	**5**	**6**	**7**	**8**	**9**	**10**	**11**	**12**	**13**	**14**	**15**	**16**	**17**	**18**	**19**	**20**	**21**	**22**	**23**	**24**	**25**	**26**
(1) Age	1																									
(2) SL	0.06	1																								
(3) PA	0.12	–0.04	1																							
(4) RAN (time)	0.01	–0.02	–0.25^∗∗^	1																						
(5) OA	0.20^∗^	–0.07	0.20^∗^	–0.06	1																					
(6) NVCog	0.17^∗^	0.04	0.49^∗∗^	–0.33^∗∗^	0.16	1																				
(7) Wacc1	0.24^∗^	0.01	0.59^∗∗^	–0.44^∗∗^	0.26^∗∗^	0.53^∗∗^	1																			
(8) NWacc1	0.14	–0.01	0.52^∗∗^	–0.26^∗∗^	0.20^∗^	0.32^∗∗^	0.75^∗∗^	1																		
(9) Wflu1	0.28^∗^	–0.05	0.50^∗∗^	–0.28^∗∗^	0.36^∗∗^	0.40^∗∗^	0.80^∗∗^	0.62^∗∗^	1																	
(10) NWflu1	0.22^∗^	–0.11	0.54^∗∗^	–0.19^∗∗^	0.30^∗∗^	0.26^∗∗^	0.74^∗∗^	0.75^∗∗^	0.76^∗∗^	1																
(11) Sp1	0.15	0.05	0.54^∗∗^	–0.23^∗∗^	0.17	0.41^∗∗^	0.78^∗∗^	0.67^∗∗^	0.73^∗∗^	0.61^∗∗^	1															
(12) Wacc2	0.18^∗^	0.02	0.60^∗∗^	–0.42^∗∗^	0.22^∗^	0.47^∗∗^	0.86^∗∗^	0.74^∗∗^	0.80^∗∗^	0.71^∗∗^	0.68^∗∗^	1														
(13) NWacc2	0.09	–0.11	0.48^∗∗^	–0.30^∗∗^	0.18^∗^	0.27^∗∗^	0.64^∗∗^	0.62^∗∗^	0.60^∗∗^	0.67^∗∗^	0.49^∗∗^	0.77^∗∗^	1													
(14) Wflu2	0.18^∗^	0.01	0.61^∗∗^	–0.36^∗∗^	0.30^∗∗^	0.45^∗∗^	0.84^∗∗^	0.71^∗∗^	0.84^∗∗^	0.73^∗∗^	0.70^∗∗^	0.89^∗∗^	0.66^∗∗^	1												
(15) NWflu2	0.07	–0.06	0.50^∗∗^	–0.27^∗∗^	0.22^∗^	0.30^∗∗^	0.68^∗∗^	0.74^∗∗^	0.67^∗∗^	0.76^∗∗^	0.53^∗∗^	0.77^∗∗^	0.79^∗∗^	0.76^∗∗^	1											
(16) Sp2	0.20^∗^	0.01	0.66^∗∗^	–0.32^∗∗^	0.32^∗∗^	0.45^∗∗^	0.83^∗∗^	0.74^∗∗^	0.80^∗∗^	0.73^∗∗^	0.78^∗∗^	0.89^∗∗^	0.74^∗∗^	0.88^∗∗^	0.76^∗∗^	1										
(17) Wacc3	0.12	–0.01	0.59^∗∗^	–0.41^∗∗^	0.15	0.45^∗∗^	0.76^∗∗^	0.67^∗∗^	0.69^∗∗^	0.62^∗∗^	0.57^∗∗^	0.86^∗∗^	0.75^∗∗^	0.84^∗∗^	0.77^∗∗^	0.81^∗∗^	1									
(18) NWacc3	0.1	–0.09	0.47^∗∗^	–0.33^∗∗^	0.09	0.37^∗∗^	0.68^∗∗^	0.58^∗∗^	0.47^∗∗^	0.53^∗∗^	0.41^∗∗^	0.69^∗∗^	0.74^∗∗^	0.64^∗∗^	0.68^∗∗^	0.70^∗∗^	0.83^∗∗^	1								
(19) Wflu3	0.02	–0.03	0.52^∗∗^	–0.39^∗∗^	0.21^∗^	0.42^∗∗^	0.72^∗∗^	0.61^∗∗^	0.67^∗∗^	0.55^∗∗^	0.56^∗∗^	0.81^∗∗^	0.66^∗∗^	0.82^∗∗^	0.73^∗∗^	0.80^∗∗^	0.90^∗∗^	0.74^∗∗^	1							
(20) NWflu3	0.05	–0.14	0.42^∗∗^	–0.39^∗∗^	0.18^∗^	0.26^∗∗^	0.61^∗∗^	0.61^∗∗^	0.58^∗∗^	0.63^∗∗^	0.42^∗∗^	0.71^∗∗^	0.70^∗∗^	0.72^∗∗^	0.77^∗∗^	0.70^∗∗^	0.84^∗∗^	0.82^∗∗^	0.84^∗∗^	1						
(21) Sp3	0.13	0.02	0.57^∗∗^	–0.37^∗∗^	0.19^∗^	0.47^∗∗^	0.75^∗∗^	0.63^∗∗^	0.67^∗∗^	0.58^∗∗^	0.62^∗∗^	0.85^∗∗^	0.71^∗∗^	0.81^∗∗^	0.72^∗∗^	0.85^∗∗^	0.89^∗∗^	0.81^∗∗^	0.84^∗∗^	0.75^∗∗^	1					
(22) Wacc4	–0.1	–0.01	0.47^∗∗^	–0.29^∗∗^	0.05	0.38^∗∗^	0.54^∗∗^	0.52^∗∗^	0.47^∗∗^	0.41^∗∗^	0.42^∗∗^	0.64^∗∗^	0.55^∗∗^	0.61^∗∗^	0.54^∗∗^	0.63^∗∗^	0.78^∗∗^	0.71^∗∗^	0.81^∗∗^	0.72^∗∗^	0.72^∗∗^	1				
(23) NWacc4	–0.06	–0.02	0.42^∗∗^	–0.27^∗∗^	0.08	0.37^∗∗^	0.48^∗∗^	0.53^∗∗^	0.40^∗∗^	0.44^∗∗^	0.33^∗^	0.59^∗∗^	0.62^∗∗^	0.53^∗∗^	0.56^∗∗^	0.57^∗∗^	0.76^∗∗^	0.82^∗∗^	0.73^∗∗^	0.73^∗∗^	0.70^∗∗^	0.86^∗∗^	1			
(24) Wflu4	–0.08	0.02	0.47^∗∗^	–0.37^∗∗^	0.08	0.44^∗∗^	0.58^∗∗^	0.49^∗∗^	0.50^∗∗^	0.39^∗∗^	0.41^∗∗^	0.65^∗∗^	0.52^∗∗^	0.67^∗∗^	0.53^∗∗^	0.64^∗∗^	0.80^∗∗^	0.74^∗∗^	0.86^∗∗^	0.72^∗∗^	0.76^∗∗^	0.91^∗∗^	0.84^∗∗^	1		1
(25) NWflu4	–0.08	–0.07	0.43^∗∗^	–0.32^∗∗^	0.16	0.32^∗∗^	0.56^∗∗^	0.57^∗∗^	0.53^∗∗^	0.52^∗∗^	0.36^∗∗^	0.63^∗∗^	0.66^∗∗^	0.62^∗∗^	0.63^∗∗^	0.62^∗∗^	0.79^∗∗^	0.79^∗∗^	0.80^∗∗^	0.82^∗∗^	0.70^∗∗^	0.83^∗∗^	0.86^∗∗^	0.86^∗∗^	1	
(26) Sp4	0.04	–0.02	0.54^∗∗^	–0.36^∗∗^	0.16	0.47^∗∗^	0.69^∗∗^	0.62^∗∗^	0.60^∗∗^	0.52^∗∗^	0.57^∗∗^	0.79^∗∗^	0.67^∗∗^	0.77^∗∗^	0.65^∗∗^	0.80^∗∗^	0.86^∗∗^	0.80^∗∗^	0.87^∗∗^	0.76^∗∗^	0.90^∗∗^	0.84^∗∗^	0.80^∗∗^	0.86^∗∗^	0.79^∗∗^	1

*SL = Statistical Learning; PA = Phonological Awareness (CTOPP-2 Elision); RAN (time) = Rapid Symbol Naming (CTOPP-2 RAN letters) response time; OA = Orthographic Awareness; NVCog = BAS-3 Non-verbal Cognition; Wacc1, Wacc2, Wacc3, Wacc4 = Word Accuracy (WJIII Word Identification) at Time 1, 2, 3, and 4, respectively; NWacc1, NWacc2, NWacc3, NWacc4 = Non-word Accuracy (WJIII Word Attack) at Time 1, 2, 3, and 4, respectively; Wflu1, Wflu2, Wflu3, Wflu4 = Word Fluency (TOWRE-2 sight word reading) at Time 1, 2, 3, and 4, respectively; NWflu1, NWflu2, NWflu3, NWflu4 = Non-word Fluency (TOWRE-2 phonemic decoding) at Time 1, 2, 3, and 4, respectively; Sp1, Sp2, Sp3, Sp4 = Spelling (BAS-3) at Time 1, 2, 3, and 4, respectively. ^∗^*p* < 0.05, ^∗∗^*p* < 0.01.*

### Research Question 1

To address the first research question regarding the optimal grain size for teaching struggling readers, mixed effects linear regression models were run separately for each outcome variable using the lmer package in R (R Studio Version 1.1.442). Participants were entered as a random variable, and the interaction of intervention group and time were entered as fixed variables. Age and non-verbal cognitive ability scores at baseline were entered as covariates in all of the models. The analysis was first conducted with data from *Phase 1* (for training grapheme–phoneme accuracy), including the pre-test and mid-test scores. This analysis compares the outcomes for a lexical focus, in the word group, versus a sublexical focus, in the other two groups. Then, the data across *Phase 2* (for training spelling-sound fluency) were analyzed, including pre-test, mid-test, and post-test scores. These analyses contrast the different phoneme-, rime- and word-unit level foci of the respective interventions. Finally, to examine the duration of effects, an analysis of post-test to follow-up data was undertaken.

#### Phase 1

Comparing performance of the intervention groups over time, from pre-test to mid-test, showed main effects where performance improved over time in each dependent variable [word reading accuracy, *t*(138.44) = 11.76, *p* < 0.001; decoding accuracy, *t*(143.17) = 6.81, *p* < 0.001; word reading fluency, *t*(137.68) = 7.98, *p* < 0.001; decoding fluency *t*(137.76) = 8.54, *p* < 0.001; spelling, *t*(131.91) = 10.67, *p* < 0.001]. None of the time by intervention group interactions were significant for this treatment phase (refer to Table 5).

**TABLE 5:** Coefficients (standard errors) for mixed effects models of word reading and decoding accuracy, fluency, and spelling from phases 1 and 2 and follow-up of the intervention.

**Fixed effects**	**Word reading accuracy *estimate (SE)***	**Decoding accuracy *estimate (SE)***	**Word reading fluency *estimate (SE)***	**Decoding fluency *estimate (SE)***	**Spelling *estimate (SE)***
**Phase 1 (pre-mid test)**					
Intercept	−1.194(1.469)	−1.282(1.504)	−0.467(0.834)	−0.499(0.674)	−3.731(1.268)**
Tx2	−0.121(0.215)	−0.121(0.220)	−0.098(0.122)	−0.189(0.099)	−0.188(0.180)
Tx3	0.159 (0.215)	0.207 (0.221)	0.021 (0.123)	0.017 (0.099)	0.174 (0.0181)
Time	0.577(0.135)***	0.575(0.126)***	0.325(0.048)***	0.233(0.042)***	0.551(0.067)***
Age	0.042(0.018)*	0.043(0.019)*	−0.015(0.010)	−0.005(0.008)	0.050(0.016)**
NV Ability	−0.007(0.003)*	−0.007(0.003)*	−0.004(0.002)**	−0.003(0.001)	−0.004(0.002)
Tx2: Time	−0.105(0.191)	−0.105(0.177)	0.019 (0.067)	0.032 (0.059)	−0.115(0.094)
Tx3: Time	−0.211(0.190)	−0.144(0.177)	0.075 (0.068)	0.081 (0.059)	−0.080(0.094)
**Phase 2 (pre-mid-post test)**					
Intercept	−0.195(0.795)	−0.412(1.899)	0.180 (0.952)	−0.130(0.747)	−2.58(1.054)*
Tx2	−0.040(0.116)	−0.427(0.276)	−0.135(0.139)	−0.190(0.110)	−0.151(0.150)
Tx3	0.095 (0.117)	−0.030(0.277)	0.077 (0.140)	0.035 (0.110)	0.127 (0.150)
Time	0.688(0.048)***	1.664(0.211)***	0.634(0.065)***	0.549(0.055)***	0.953(0.700)***
Age	0.025(0.010)*	0.042 (0.024)	−0.019(0.012)	−0.007(0.009)	0.036(0.013)**
NV Ability	−0.006(0.002)***	−0.009(0.004)*	−0.006(0.002)**	−0.003(0.001)*	−0.005(0.002)*
Tx2: Time	−0.130(0.067)	−0.702(0.296)*	−0.065(0.090)	0.005 (0.076)	−0.080(0.098)
Tx3: Time	−0.039(0.067)	−0.514(0.296)	0.145 (0.092)	0.073 (0.077)	−0.033(0.098)
**Post-followup test**					
Intercept	0.163 (1.272)	0.637 (3.477)	−0.226(1.673)	0.089 (1.171)	−2.098(1.409)
Tx2	−0.187(0.180)	−0.838(0.491)	−0.163(0.237)	−0.183(0.166)	−0.118(0.199)
Tx3	0.086 (0.180)	−0.114(0.491)	0.220 (0.237)	0.105 (0.166)	0.151 (0.199)
Time	0.193(0.005)***	−0.067(0.209)	−0.574(0.084)***	−0.367(0.056)***	0.048 (0.043)
Age	0.031 (0.016)	0.048 (0.044)	−0.009(0.021)	−0.005(0.015)	0.032 (0.018)
NV Ability	−0.008(0.002)**	−0.013(0.006)*	−0.012(0.003)***	−0.007(0.002)**	−0.008(0.003)**
Tx2: Time	0.001 (0.076)	0.327 (0.296)	0.087 (0.120)	0.042 (0.078)	0.104 (0.060)
Tx3: Time	0.094 (0.076)	0.464 (0.296)	0.129 (0.119)	0.071 (0.078)	0.099 (0.060)

*Word reading accuracy and decoding accuracy are grade equivalent scores from the WJIII, word reading fluency and decoding fluency are norm-referenced z-scores, and BAS Spelling are scaled scores. NV Ability = non-verbal ability. Tx2 = rime-level compared to phoneme-level intervention effects; Tx3 = word-level compared with phoneme-level intervention effects. ^∗∗∗^*p* < 0.001; ^∗∗^*p* < 0.01; ^∗^*p* < 0.05.*

#### Phase 2

**FIGURE 1:**
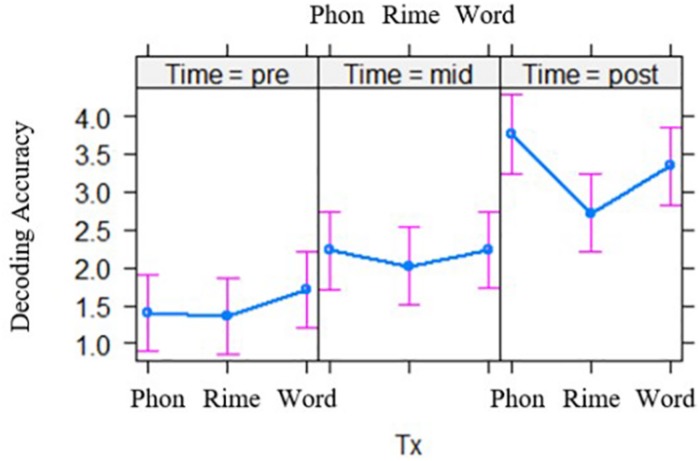
Effects of intervention groups (Tx), on decoding accuracy across two phases of intervention. Phon = phoneme-level, Rime = rime-level, Word = word-level intervention conditions. Group differences are shown across pre-test **(left)**, mid-test **(middle)**, and post-test **(right)**.

Models with three time points, pre-test, mid-test and post-test, again showed significant main effects of time for each of the dependent variables [word reading accuracy, *t*(272.22) = 14.39, *p* < 0.001; decoding accuracy, *t*(279.53) = 7.88, *p* < 0.001; word reading fluency, *t*(268.55) = 9.70 *p* < 0.001; decoding fluency *t*(268.86) = 10.04, *p* < 0.001; spelling, *t*(256.46) = 13.62, *p* < 0.001]. In addition, the time by intervention group interaction was significant for decoding accuracy, *t*(278.43) = −2.37, *p* = 0.018 (see Figure 1). *Post hoc* pairwise comparisons showed that the phoneme-level and rime-level intervention groups differed in their slopes for performance over time, *t*(281) = 2.37, *p* = 0.048 (with Tukey adjustment). There was also a trend for the interaction of intervention group by time for word reading accuracy, *t*(271.62) = −1.94, *p* = 0.053. However, *post hoc* contrasts showed no differences between the intervention groups in terms of word reading performance over time (*p*’s > 0.05). The time by intervention group effects were not significant for the other measures (see Table 5).

#### Follow-Up

Finally, after the intervention at post-test, all students continued to show growth in word reading accuracy, which showed main effects of time from post-test to follow-up, *t*(126.9) = 3.161, *p* < 0.001. Word reading fluency and decoding fluency decreased over this time period, however, *t*(126.38) = −6.808, *p* < 0.001; *t*(126.42) = −6.618, *p* < 0.001, meaning these children were not keeping up with the progress of their peers when they had entered the next grade in school. There was no difference over this time period in decoding accuracy scores, or spelling scores, although spelling did reveal a trend for intervention group (the rime-level compared with the phoneme-level group) over time effects, *t*(125.25) = 1.73, *p* = 0.085 (Table 5).

#### Summary

After the first 7 weeks of the interventions (phase 1), each intervention group showed improved scores on each of the five outcome measures (word reading and decoding, fluency for word reading and decoding, and spelling), with no evident advantage for either a word-level or subword-level focused approach. After the second 7 weeks of intervention (phase 2), all groups again showed similar levels of improvement on outcomes, except that decoding accuracy showed greater improvement for the phoneme-level intervention group compared to the rime-level group. Finally, 3 months after the intervention, for all groups word reading continued to improve, while performance on fluency measures lagged behind typical peers. Similar to previous research, fluency skills are the most difficult to remediate (Metsala and David, 2017).

### Research Question 2

To address the second research question regarding effects of individual characteristics of struggling readers, and whether these moderate the effect of intervention, we added to the models the baseline measures of individual performance to examine possible effects and interactions with learning outcomes. Orthographic awareness and rapid symbol naming were included as covariates, along with age and non-verbal ability. Additionally, phonological awareness, statistical learning, and the child’s mother tongue language group were included in models, each as interaction terms with intervention group by time. Just as in the analysis for research question 1, we first ran mixed models with data from *Phase 1* of the intervention, including the pre-test and mid-test scores. Then, the data across *Phase 2* of the intervention were analyzed, including pre-test, mid-test, and post-test scores. Finally, to examine the duration of effects, an analysis of post-test to follow-up data was undertaken.

#### Phase 1

The mixed regression models on pre-test to mid-test data for each of the outcomes are reported in Table 6A. We focus on the interaction effects of intervention group over time by (1) phonological awareness, (2) statistical learning, and (3) bilingual language group. At Phase 1, the word-level intervention group, which involves lexical level processing, is compared to a sublexical focus in the other interventions.

**TABLE 6A:** Coefficients (standard errors) for mixed effects models including baseline measure moderators of word reading and decoding accuracy, fluency, and spelling from Phase 1 of the intervention.

**Fixed effects**	**Word reading accuracy *estimate (SE)***	**Decoding accuracy *estimate (SE)***	**Word reading fluency *estimate (SE)***	**Decoding fluency *estimate (SE)***	**Spelling *estimate (SE)***
**Phase 1 (pre-mid test)**					
Intercept	−0.154(0.606)	−0.584(1.46)	−0.661(0.833)	−0.529(0.700)	−2.942(1.005)**
Tx2	0.275 (0.208)	0.197 (0.500)	0.244 (0.286)	−0.035(0.24)	0.124 (0.339)
Tx3	0.109 (0.188)	0.609 (0.451)	0.222 (0.258)	0.032 (0.217)	0.043 (0.308)
Time.L	0.168(0.074)*	0.721(0.280)*	0.185(0.094).	0.101(0.097).	0.254(0.134).
PA	0.050(0.014)***	0.119(0.035)***	0.080(0.02)***	0.056(0.017)***	0.101(0.024)***
SL	−0.034(0.038)	−0.195(0.093)*	−0.038(0.052)	−0.048(0.044)	−0.046(0.062)
Lang_M	−0.123(0.118)	−0.438(0.284)	−0.284(0.161).	−0.059(0.136).	−0.271(0.192)
Lang_T	−0.28(0.24)	−0.741(0.576)	−0.328(0.33)	−0.107(0.277)	−0.582(0.387)
Age	0.012 (0.007)	0.013 (0.018)	−0.023(0.01)*	−0.012(0.009)*	0.022(0.013).
NV Ability	0.008 (0.007)	0.021 (0.016)	−0.009(0.009)	−0.003(0.008)	0.007 (0.011)
OA	0.014(0.008).	0.040(0.019)*	0.024(0.011)*	0.011(0.009)*	0.035(0.013)**
RAN	−0.014(0.007)*	−0.028(0.017).	0.005 (0.009)	0.001 (0.008)	−0.011(0.011)
Tx2: Time	0.102 (0.118)	−0.400(0.447)	0.026 (0.149)	0.181 (0.154)	0.208 (0.221)
Tx3: Time	0.01 (0.106)	−0.275(0.402)	−0.181(0.134)	0.25 (0.139)	0.127 (0.206)
Tx2: PA	−0.04(0.018)*	−0.093(0.042)*	−0.056(0.024)*	−0.026(0.02)*	−0.062(0.029)*
Tx3: PA	−0.007(0.015)	−0.110(0.035)**	−0.035(0.020).	−0.015(0.017).	0.001 (0.025)
Time: PA	0.01 (0.006)	0.062(0.024)*	0.017(0.008)*	0.014(0.008)*	0.054(0.012)***
Tx2: SL	0.050 (0.052)	0.220(0.126).	0.074 (0.072)	0.052 (0.06)	0.050 (0.084)
Tx3: SL	0.076 (0.062)	0.301(0.15)*	0.002 (0.086)	0.015 (0.072)	0.141 (0.101)
Time: SL	−0.020(0.023)	−0.214(0.087)*	−0.043(0.030)	0.019 (0.03)	−0.062(0.042)
Tx2: EM	−0.113(0.178)	0.318 (0.428)	−0.06(0.244)	−0.007(0.206)	0.153 (0.289)
Tx3: EM	−0.107(0.173)	0.203 (0.418)	0.041 (0.238)	0.068 (0.2)	−0.089(0.282)
Tx2: ET	0.444 (0.295)	1.074 (0.709)	0.456 (0.405)	0.207 (0.34)	0.909(0.475).
Tx3: ET	0.153 (0.301)	0.645 (0.722)	0.456 (0.414)	0.276 (0.347)	0.546 (0.485)
Time: EM	0.061 (0.069)	−0.805(0.261)**	0.098 (0.088)	0.026 (0.09)	−0.115(0.127)
Time: ET	−0.003(0.136)	−0.616(0.518)	−0.028(0.172)	−0.149(0.178)	0.032 (0.247)
Tx2: Time: PA	−0.007(0.01)	−0.044(0.038)	0.003 (0.013)	−0.008(0.013)	−0.043(0.02)*
Tx3: Time: PA	−0.003(0.008)	−0.058(0.032).	0.027(0.011)*	−0.015(0.011)*	−0.026(0.018)
Tx2: Time: SL	0.027 (0.031)	0.189 (0.117)	0.084(0.039)*	0.015(0.041)*	0.131(0.056)*
Tx3: Time: SL	0.048 (0.035)	0.137 (0.132)	0.075(0.044).	−0.049(0.046).	−0.008(0.064)
Tx2: Time: EM	−0.171(0.102).	0.929(0.389)*	−0.133(0.13)	−0.159(0.134)	−0.237(0.191)
Tx3: Time: EM	−0.198(0.099)*	0.890(0.376)*	−0.065(0.126)	−0.044(0.13)	−0.045(0.185)
Tx2: Time: ET	−0.074(0.169)	0.76 (0.641)	−0.047(0.214)	0.056 (0.221)	0.018 (0.306)
Tx3: Time: ET	0.077 (0.172)	0.493 (0.654)	−0.025(0.218)	0.213 (0.225)	0.095 (0.312)

*Word reading accuracy and decoding accuracy are grade equivalent scores from the WJIII, word reading fluency and decoding fluency are norm-referenced z-scores, and BAS Spelling are scaled scores. NV Ability = non-verbal ability, OA = orthographic awareness, PA = phonological awareness, SL = statistical learning, Lang_M = English/Malay group, Lang_T = Egnlish/Tamil group. EM = English/Malay group vs. English/Chinese group, ET = English/Tamil group vs. English/Chinese group. Tx2 = rime-level compared to phoneme-level intervention effects; Tx3 = word-level compared with phoneme-level intervention effects. ^∗∗∗^*p* < 0.001; ^∗∗^*p* < 0.01; ^∗^*p* < 0.05.*

**FIGURE 2:**
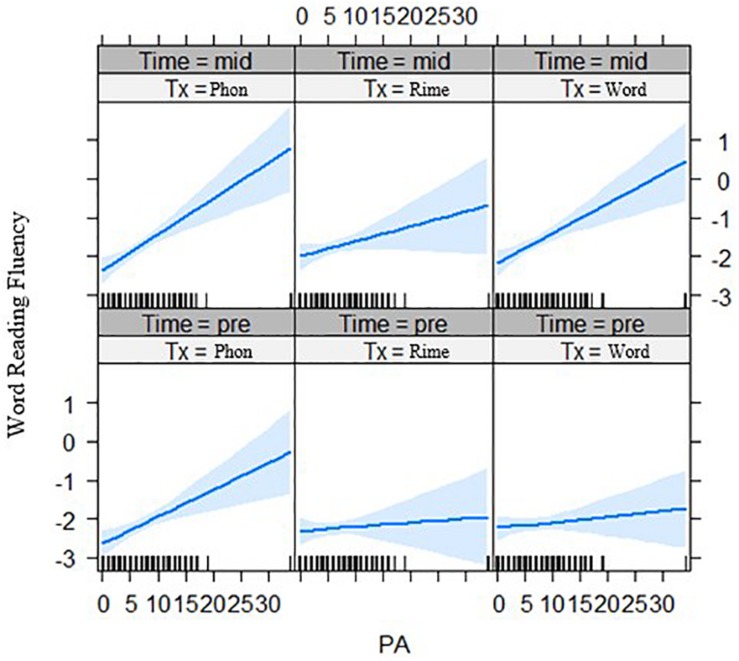
Effects of phonological awareness on word reading fluency (z-scores) for each intervention group over two time points (pre-test and mid-test). PA = phonological awareness score, Tx_Phon = phoneme-level group, Tx_Rime = rime-level group, Tx_Word = word-level intervention group.

First, for the interaction of phonological awareness by intervention group by time effects, the three-way interaction was significant for word reading fluency outcomes after the first phase of intervention, *t*(119.50) = 2.54, *p* = 0.012 (Figure 2). Generally, the relation of phonological awareness to outcomes became stronger over time for the word-level intervention group compared with the phoneme-level intervention group. Second, there were no interaction effects for statistical learning with time by intervention groups with a lexical versus sublexical unit focus.

**FIGURE 3:**
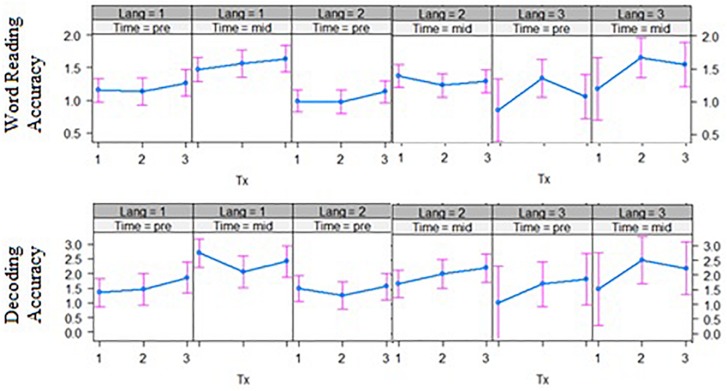
Bilingual language groups by intervention group effects over two time points for word reading accuracy **(top)** and decoding accuracy **(bottom)** (grade equivalent scores). Tx1 = phoneme-level, Tx2 = rime-level, Tx3 = word-level intervention. Pre = pre-test, Mid = mid-test; Lang 1 = English and Chinese, Lang 2 = English and Malay, Lang 3 = English and Tamil bilingual groups.

Third, for the interaction of bilingual language group by intervention group by time effects, word reading accuracy outcomes after phase 1 of the intervention showed a significant three-way interaction effect, *t*(121.32) = −1.67, *p* = 0.049 (Figure 3, left panel). The English–Malay and English–Chinese bilingual individuals differed in their response to intervention at the word-level, where the former did not improve over time in the word-level group (*p* > 0.05), but the latter group did (*p* < 0.001). Also, decoding accuracy outcomes revealed a significant three-way interaction across the phoneme-level intervention versus word-level, *t*(118.84) = 2.36, *p* = 0.020, and rime-level conditions, *t*(118.71) = 2.39, *p* = 0.018 (Figure 3, right panel). In this case, the English–Chinese bilinguals benefited more from the phoneme-level intervention, while the English–Malay bilinguals benefited more from the rime-level intervention (*p*’s < 0.05). No other three-way interaction effects with bilingual language group were significant.

#### Phase 2

For the second phase of the intervention, the mixed regression models on pre-test, mid-test and post-test data for each of the outcomes are reported in Table 6B. The interaction effects of interest are reported in each section below for intervention group by time with (1) phonological awareness, (2) statistical learning, and (3) bilingual language group.

**TABLE 6B:** Coefficients (standard error) for mixed effects models including baseline measure moderators of word reading and decoding accuracy, fluency, and spelling from Phase 2 of the intervention.

**Fixed effects**	**Word reading accuracy *estimate (SE)***	**Decoding accuracy *estimate (SE)***	**Word reading fluency *estimate (SE)***	**Decoding fluency *estimate (SE)***	**Spelling *estimate (SE)***
**Phase 2 (pre-mid-post test)**					
Intercept	0.245 (0.634)	−0.699(1.681)	0.376 (0.847)	−0.123(0.705)	−1.977(0.775)
Tx2	−0.012(0.200)	0.141 (0.584)	−0.138(0.263)	−0.29(0.226)	−0.24(0.244)
Tx3	−0.008(0.19)	0.270 (0.525)	−0.073(0.252)	−0.096(0.213)	−0.127(0.232)
Time	0.255(0.11)*	0.387 (0.484)	0.101 (0.138)	0.237(0.133).	0.45 (0.146)
PA	0.021(0.01)*	0.169(0.031)***	0.030(0.012)*	0.036(0.011)**	0.04(0.012)***
SL	−0.061(0.041)	−0.268(0.109)*	−0.08(0.055)	−0.071(0.046)	−0.064(0.049)*
Lang_M	−0.061(0.125)	−0.368(0.327)	−0.267(0.167)	−0.115(0.139)	−0.229(0.15)
Lang_T	−0.477(0.252).	−1.334(0.649)*	−0.53(0.337)	−0.281(0.279)	−0.604(0.297)*
Age	0.007 (0.008)	0.006 (0.02)	−0.033(0.01)**	−0.017(0.009)*	0.012 (0.009)
NV Ability	0.022(0.005)***	0.01 (0.015)	0.013(0.007).	0.007 (0.006)	0.023 (0.006)
OA	0.024(0.004)***	0.072(0.017)***	0.025(0.006)***	0.02(0.005)***	0.031(0.006)***
RAN	−0.028(0.005)***	−0.017(0.015)	−0.018(0.007)**	−0.009(0.006)	−0.028(0.006)
Tx2: Time	0.133 (0.188)	−0.11(0.814)	0.472(0.235)*	0.192 (0.226)	0.005 (0.254)
Tx3: Time	−0.06(0.157)	−1.07(0.69)	−0.139(0.196)	0.009 (0.189)	−0.016(0.214)
Tx2: PA	−0.013(0.013)	−0.105(0.043)*	−0.015(0.017)	−0.007(0.015)	−0.008(0.017)*
Tx3: PA	−0.003(0.012)	−0.116(0.036)**	0.002 (0.015)	−0.004(0.013)	0.013(0.015)**
Time: PA	0.028(0.008)***	0.11(0.035)**	0.039(0.01)***	0.022(0.01)*	0.049(0.011)**
Tx2: SL	0.085 (0.055)	0.253(0.146).	0.15(0.074)*	0.07 (0.061)	0.085(0.066).
Tx3: SL	0.147(0.065)*	0.521(0.172)**	0.042 (0.087)	0.035 (0.072)	0.157(0.077)**
Time: SL	−0.039(0.031)	−0.26(0.137).	−0.056(0.039)	−0.014(0.038)	−0.051(0.042).
Tx2: EM	−0.063(0.185)	0.333 (0.489)	0 (0.247)	0.181 (0.206)	0.086 (0.221)
Tx3: EM	−0.158(0.184)	0.286 (0.48)	−0.022(0.246)	0.123 (0.204)	−0.138(0.219)
Tx2: ET	0.689(0.307)*	1.644(0.795)*	0.767(0.41).	0.546 (0.34)	0.805(0.363)*
Tx3: ET	0.387 (0.318)	1.189 (0.821)	0.556 (0.426)	0.419 (0.352)	0.56 (0.376)
Time: EM	0.187(0.093)*	−0.209(0.41)	0.132 (0.116)	−0.08(0.112)	−0.131(0.125)
Time: ET	−0.203(0.181)	−1.383(0.807).	−0.153(0.226)	−0.265(0.218)	−0.169(0.24).
Tx2: Time: PA	−0.017(0.014)	−0.07(0.059)	−0.04(0.017)*	−0.022(0.017)	−0.012(0.019)
Tx3: Time: PA	−0.005(0.011)	−0.018(0.049)	0.04(0.014)**	0.003 (0.014)	−0.01(0.017)
Tx2: Time: SL	0.049 (0.042)	0.161 (0.183)	0.116(0.052)*	0.01 (0.05)	0.115 (0.055)
Tx3: Time: SL	0.073 (0.049)	0.443(0.216)*	−0.008(0.062)	−0.068(0.059)	0.011(0.067)*
Tx2: Time: EM	−0.251(0.141).	0.286 (0.621)	−0.339(0.177).	0.034 (0.17)	−0.29(0.192)
Tx3: Time: EM	−0.226(0.133).	0.63 (0.59)	−0.208(0.166)	0.105 (0.16)	−0.143(0.18)
Tx2: Time: ET	0.138 (0.224)	1.254 (0.995)	0.184 (0.28)	0.382 (0.27)	0.137 (0.297)
Tx3: Time: ET	0.431(0.228).	1.546 (1.019)	−0.045(0.285)	0.341 (0.275)	0.269 (0.303)

*Word reading accuracy and decoding accuracy are grade equivalent scores from the WJIII, word reading fluency and decoding fluency are norm-referenced z-scores, and BAS Spelling are scaled scores. NV Ability = non-verbal ability, OA = orthographic awareness, PA = phonological awareness, SL = statistical learning, Lang_M = English/Malay group, Lang_T = Egnlish/Tamil group. EM = English/Malay group vs. English/Chinese group, ET = English/Tamil group vs. English/Chinese group. Tx2 = rime-level compared to phoneme-level intervention effects; Tx3 = word-level compared with phoneme-level intervention effects. ^∗∗∗^*p* < 0.001; ^∗∗^*p* < 0.01; ^∗^*p* < 0.05.*

**FIGURE 4:**
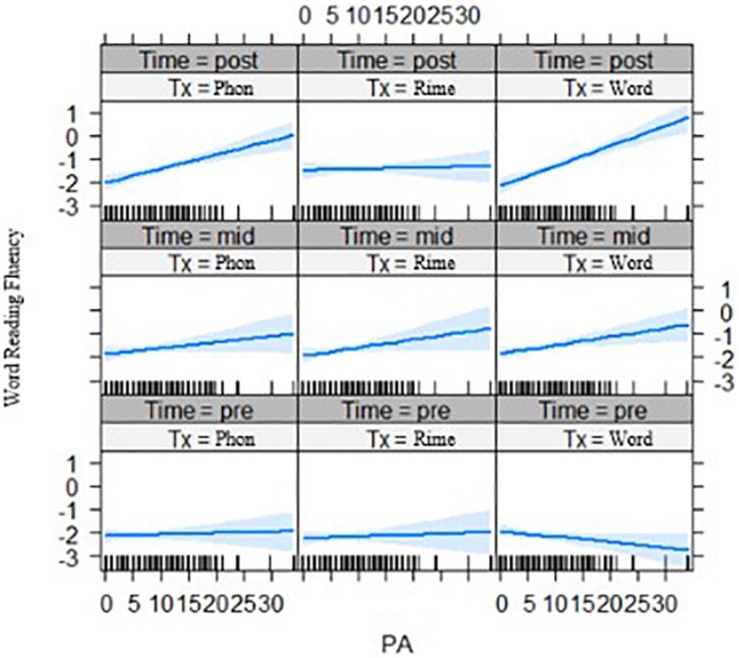
Effects of phonological awareness on word reading fluency (z-scores) for each intervention group over three time points (pre-test, mid-test, and post-test). PA = phonological awareness score, Tx_Phon = phoneme-level group, Tx_Rime = rime-level group, Tx_Word = word-level intervention group.

First, for the phonological awareness by group by time effects, the three-way interaction was significant for word reading fluency outcomes after the two phases of intervention, for both the rime-level vs. phoneme-level intervention groups, *t*(250.8) = −2.32, *p* = 0.021, and for the word-level vs. phoneme-level intervention groups, *t*(246.2) = 2.77, *p* = 0.006 (see Figure 4). Phonological awareness had less of an effect on outcomes for the rime-level intervention group. On the other hand, phonological awareness showed stronger effects on outcomes for the word-level intervention group.

**FIGURE 5:**
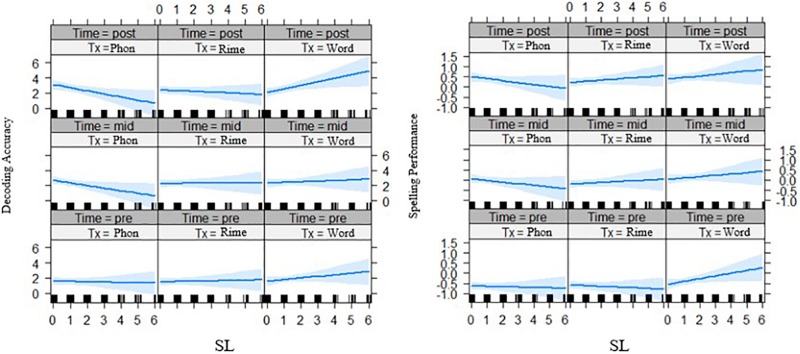
Effects of statistical learning by intervention groups on decoding accuracy **(left)** (grade equivalent scores) and spelling **(right)** (scaled scores) at three time points (pre-test, mid-test, post-test). SL = statistical learning score, Tx_Phon = phoneme-level group, Tx_Rime = rime-level group, Tx_Word = word-level intervention group.

**FIGURE 6:**
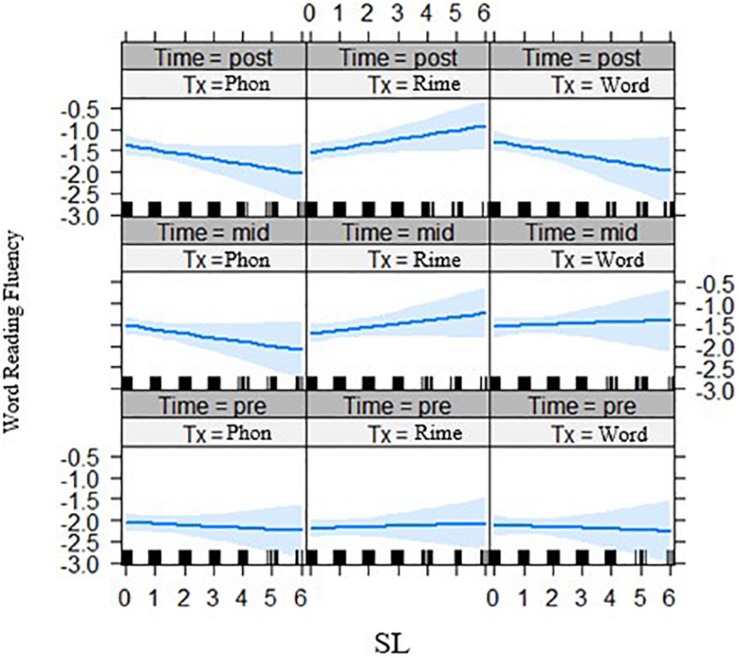
Effects of statistical learning by intervention groups on word reading fluency (z-scores) across three time points (pre-test, mid-test, post-test). SL = statistical learning score, Tx_Phon = phoneme-level group, Tx_Rime = rime-level group, Tx_Word = word-level intervention group.

**FIGURE 7:**
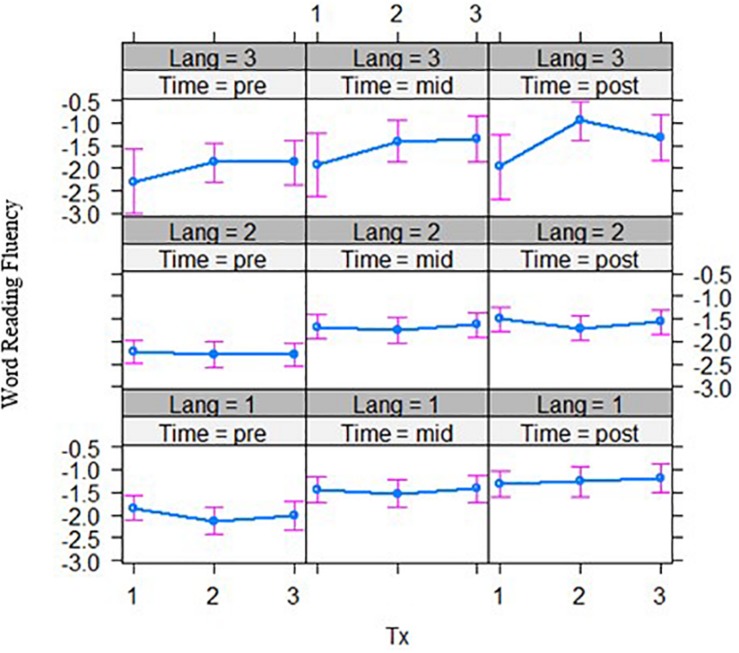
Bilingual language groups by intervention group effects on word reading fluency (z-scores) over three time points. Lang 1 = English and Chinese, Lang 2 = English and Malay, Lang 3 = English and Tamil bilingual groups; Tx1 = phoneme-level, Tx2 = rime-level, Tx3 = word-level intervention. Pre = pre-test, Mid = mid-test, Post = post-test.

Second for the interaction of statistical learning by intervention group by time, there was a significant three-way interaction effect for decoding accuracy, *t*(261.02) = 2.05, *p* = 0.041 (Figure 5, left panel) across phase 2 of the intervention, and for spelling outcomes *t*(237.85) = 2.08, *p* = 0.039 (Figure 5, right panel). In both cases, it appears that with lower statistical learning there were better outcomes for those in the phoneme-level intervention group over time. In addition, the three-way interaction of statistical learning by intervention group by time was significant for word reading fluency, *t*(235.0) = 2.24, *p* = 0.027, where there were significant interactions on performance for the rime-level compared with phoneme-level intervention groups (Figure 6). Third, for differences across the bilingual language groups there was only a marginal three-way interaction on word reading fluency, *t*(232.0) = −1.92 *p* = 0.056 (Figure 7), indicating that the English–Malay bilingual individuals did not benefit as much from the rime-level intervention as did the English–Chinese bilinguals.

#### Follow-Up

The data from post-test to follow-up test revealed that a three-way interaction of time by intervention group by statistical learning was significant for spelling scores, *t*(112.8) = 2.27, *p* = 0.025, such that the rime-level intervention yielded better outcomes over time for those with higher statistical learning. Also, word reading fluency showed a significant effect of time by intervention group by bilingual language group, *t*(114.5) = 2.174, *p* = 0.032, with the word-level intervention yielding improved outcomes over time for the English–Malay bilingual individuals compared with the English–Chinese bilinguals (Table 6C).

**TABLE 6C:** Coefficients for mixed effects models of Word reading, Decoding and Spelling from Post-Followup of the intervention including baseline measure moderators.

**Fixed effects**	**Word reading accuracy *estimate (SE)***	**Decoding accuracy *estimate (SE)***	**Word reading fluency *estimate (SE)***	**Decoding fluency *estimate (SE)***	**Spelling *estimate (SE)***
**Post-followup test**					
Intercept	−0.991(0.954)	−2.614(3.144)	−1.73(1.307)	−1.289(1.009)	−2.948(0.97)**
Tx2	0.02 (0.374)	0.134 (1.231)	0.36 (0.511)	−0.162(0.395)	−0.165(0.379)
Tx3	−0.125(0.314)	−0.974(1.035)	−0.225(0.43)	−0.341(0.332)	−0.138(0.319)
Time.L	0.027 (0.138)	−0.127(0.549)	−1.052(0.193)***	−0.55(0.14)***	0.04 (0.111)
PA	0.057(0.015)***	0.205(0.049)***	0.08(0.021)***	0.06(0.016)***	0.062(0.015)**
SL	−0.06(0.062)	−0.269(0.204)	−0.113(0.085)	−0.08(0.066)	−0.109(0.063).
Lang_M	0.141 (0.181)	0.606 (0.594)	−0.101(0.248)	0.003 (0.191)	−0.201(0.184)
Lang_T	−0.702(0.342)*	−1.432(1.123)	−0.868(0.467).	−0.528(0.361)	−0.694(0.347)*
Age	0.014 (0.011)	0.017 (0.037)	−0.027(0.015).	−0.012(0.012)	0.012 (0.011)
NV Ability	0.02(0.007)**	0.002 (0.023)	0.013 (0.009)	0.002 (0.007)	0.016(0.007)*
OA	0.054(0.009)***	0.11(0.029)***	0.069(0.012)***	0.041(0.009)***	0.058(0.009)**
RAN	−0.025(0.007)***	−0.007(0.023)	−0.02(0.009)*	−0.005(0.007)	−0.022(0.007)**
Tx2: Time	−0.049(0.238)	−0.055(0.946)	0.196 (0.332)	0.052 (0.242)	0.232 (0.191)
Tx3: Time	0.01 (0.198)	−0.265(0.787)	−0.092(0.277)	−0.142(0.201)	−0.009(0.159)
Tx2: PA	−0.028(0.023)	−0.089(0.076)	−0.058(0.032).	−0.022(0.024)	−0.01(0.023)
Tx3: PA	0.012 (0.02)	−0.017(0.065)	0.029 (0.027)	0.025 (0.021)	0.018 (0.02)
Time: PA	0.019(0.008)*	−0.05(0.033)	0.05(0.012)***	0.011 (0.008)	0.008 (0.007)
Tx2: SL	0.138(0.08).	0.33 (0.264)	0.303(0.11)**	0.112 (0.085)	0.186(0.082)*
Tx3: SL	0.118 (0.099)	0.727(0.327)*	0.092 (0.136)	−0.015(0.105)	0.187(0.101).
Time: SL	0.006 (0.038)	0.157 (0.152)	0 (0.053)	−0.006(0.039)	−0.06(0.031).
Tx2: EM	−0.114(0.271)	−0.536(0.893)	−0.332(0.372)	0.082 (0.287)	−0.089(0.276)
Tx3: EM	−0.318(0.264)	−0.015(0.868)	−0.173(0.361)	0.131 (0.279)	−0.371(0.269)
Tx2: ET	0.931(0.422)*	1.575 (1.388)	0.948 (0.577)	1.061(0.445)*	0.837(0.428).
Tx3: ET	0.606 (0.437)	1.422 (1.438)	0.592 (0.597)	0.638 (0.461)	0.65 (0.443)
Time: EM	−0.09(0.11)	0.911(0.439)*	−0.161(0.155)	0.152 (0.113)	−0.041(0.089)
Time: ET	−0.238(0.213)	1.031 (0.848)	−0.501(0.297).	−0.218(0.216)	−0.131(0.171)
Tx2: Time: PA	−0.01(0.015)	0.069 (0.059)	−0.018(0.021)	−0.002(0.015)	−0.022(0.012).
Tx3: Time: PA	0.003 (0.013)	0.084(0.05).	−0.019(0.018)	0.009 (0.013)	0.002 (0.01)
Tx2: Time: SL	0.05 (0.049)	0.059 (0.196)	0.099 (0.069)	0.077 (0.05)	0.09(0.04)*
Tx3: Time: SL	−0.018(0.059)	−0.095(0.237)	0.138 (0.083)	0.017 (0.06)	0.054 (0.049)
Tx2: Time: EM	0.206 (0.171)	−0.886(0.682)	0.039 (0.24)	−0.223(0.175)	0.037 (0.138)
Tx3: Time: EM	0.123 (0.159)	−0.443(0.635)	0.487(0.224)*	0.096 (0.163)	0.063 (0.129)
Tx2: Time: ET	0.346 (0.266)	−0.925(1.06)	0.296 (0.372)	0.427 (0.271)	0.187 (0.214)
Tx3: Time: ET	−0.003(0.279)	−1.091(1.108)	0.427 (0.389)	0.235 (0.283)	0.148 (0.224)

*Word reading accuracy and decoding accuracy are grade equivalent scores from the WJIII, word reading fluency and decoding fluency are norm-referenced z-scores, and BAS Spelling are scaled scores. NV Ability = non-verbal ability, OA = orthographic awareness, PA = phonological awareness, SL = statistical learning, Lang_M = English/Malay group, Lang_T = Egnlish/Tamil group. EM = English/Malay group vs. English/Chinese group, ET = English/Tamil group vs. English/Chinese group. Tx2 = rime-level compared to phoneme-level intervention effects; Tx3 = word-level compared with phoneme-level intervention effects. ^∗∗∗^*p* < 0.001; ^∗∗^*p* < 0.01; ^∗^*p* < 0.05.*

#### Summary

Individual differences in phonological awareness, statistical learning, and bilingual groups moderated outcomes in a way that interacted with the intervention approach. After phase 1, in the word-level intervention, those with better phonological awareness benefited more than those with poorer phonological awareness when it comes to word reading fluency. Word-level intervention affected word reading accuracy for English–Chinese bilinguals but not for English–Malay bilinguals, whereas for decoding accuracy these bilingual groups benefited only in the phoneme-level intervention for the former, and the rime-level intervention for the latter group.

After both phases of the intervention, word reading fluency outcomes were moderated by phonological awareness, with a continued positive effect in the word-level intervention and no effect in the rime-level intervention. Word reading fluency, along with decoding accuracy and spelling outcomes, were moderated by statistical learning. In this case, those with poor statistical learning benefited from the phoneme-level intervention, whereas those with high statistical learning seemed to gain more from the word-level intervention on the accuracy measures, and from rime-level intervention on the fluency measure. Effects of statistical learning by intervention persisted for spelling scores at the follow-up, with the rime-intervention group continuing to improve compared to the phoneme-level group.

#### On-Line Measures

**FIGURE 8:**
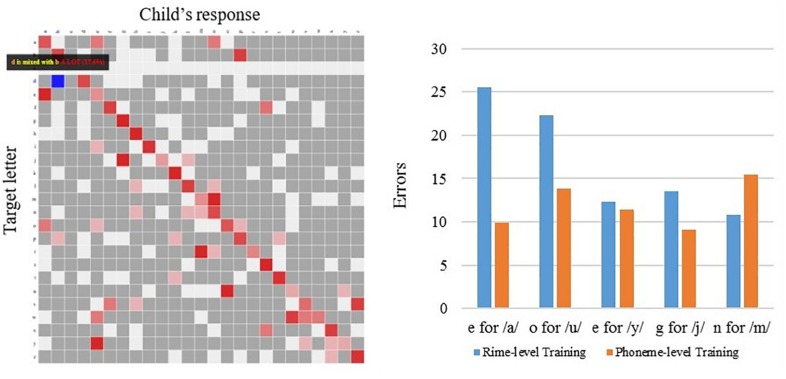
Letter confusions collected on-line, while students played *Grapholearn* app (Phase 2), across intervention groups. **(Left)** Shows example of a letter confusion matrix, where red shading indictates the proportion of times a letter (x-axis) was confused for a target letter (y-axis). **(Right)** Shows summary across children in the rime-level intervention group (blue bars) and the phoneme-level intervention group (orange bars). Bars indicate the proportion of confusion errors across different letters, with an incorrect response (‘e’) to a given sound (/a/).

In addition to the pre-intervention and post-intervention measures, an additional benefit of technology-based interventions is the capacity to capture on-line performance, in a trial by trial fashion, as the student engages with the application and performs the activities. For example, from the *Grapholearn* app, the program collects students’ data across activity levels, and compiles confusion matrices for the graphemes selected by the student, versus the grapheme that is the correct response. This results in a matrix, as shown below in Figure 8 (left panel). This plot shows how certain phonemes represented by a letter (on the y-axis) are misidentified with an incorrect corresponding letter (across the x-axis); for example, an expected response of the letter ‘i’ (in the 9^th^ row) is often mistaken with a response of ‘e’ (in the 5^th^ column). The high proportion of these incorrect responses is indicated with a red block, and these red blocks off the diagonal represent these high occurrence confusions. The summary of these higher incidence confusions are tallied across individuals within the intervention groups, and presented in the Figure 8, right panel. Examining the confusion matrices across the intervention groups that used the *Grapholearn* app – the phoneme-level and rime-level groups – the rime-level group appears to show more vowel confusions for corresponding letters for the sounds of/a/and/o/than the phoneme-level group made. On the other hand, the phoneme-level group made more consonant confusions for /m/ (Figure 8, right panel).

**FIGURE 9:**
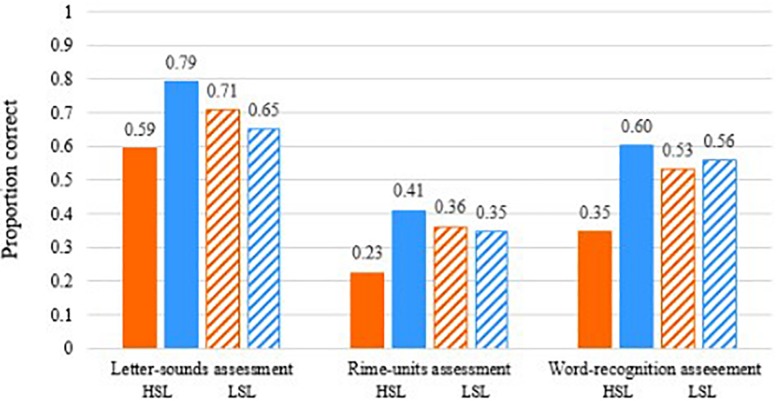
On-line data from *Grapholearn* mini-assessments for groups of students in the phoneme-level intervention group (orange bars) and the rime-level intervention group (blue bars). HSL = High statistical learners (solid bars), LSL = Low statistical learners (striped bars). Performance (proportion correct) on letter-sound items **(left)**, rime items **(middle)**, and word items **(right)**.

However, given the interactions of intervention types with individual differences, noted above in the results on the pre-test and post-test outcome measures, we also examine the performance on the app tasks for those individuals considered as low on statistical learning (e.g., scoring at chance) compared with those scoring highest on statistical learning (including the highest scorers in the groups). In Figure 9, we examined children’s *Grapholearn* scores on the mini-assessments conducted throughout the intervention. Here, the high statistical learners (solid bars) showed a difference between the phoneme-level and rime-level intervention performances. Phoneme-level intervention shows poorer performance on the assessment tasks (orange bar), at all unit levels (Phoneme, Rime and Word identification and matching), and compared with all the other groups of individuals. This fits with our expectation that stronger statistical learning may enable one to learn better in terms of orthographic patterns, rather than individual letters.

## Discussion

We examined the effects of technology-mediated reading interventions focused at different unit-sizes posited as optimal input for learning to read English. These included interventions focused at the level of either the phoneme, rime or whole word unit for struggling learners. Our findings differ from previous studies, which showed sublexical (syllable or onset-rime) unit benefits over lexical (word-level) units (Olson and Wise, 1992; Ecalle et al., 2009) or rime-level benefits over phoneme-level units (Kyle et al., 2013). In general, only decoding accuracy showed an overall effect where phoneme-level intervention yielded better growth over time than the rime-level intervention, while word-level intervention did not differ.

To summarize, the reading and spelling outcomes increased across all groups across two phases of intervention with tablet-based gamelike apps. After the intervention, at 3 months’ followup, children’s word reading continued to improve, while spelling and decoding skills were maintained, but their reading fluency declined relative to peers. Thus, the intervention program of the school plus the applications in this project may have helped them continue to learn to read words, but did not improve their fluency for word reading. The set of findings did not strictly conform to our hypotheses. First, we hypothesized that the word-level group would show least progress, as lexical processing would be less efficient than learning sublexical GPC patterns that could be applied across novel words or pseudowords. This was not the case, as all intervention groups showed similar progress in the first phase of the intervention. Generally, there was no clear pattern of a particular advantage for teaching at any given unit level or “grain size.”

When we took into consideration possible moderators of intervention effects, we observed different patterns of influence depending on the outcome measure examined. Moderators of phonological awareness, statistical learning, and bilingual language group each showed some interaction with intervention type. Children’s phonological awareness moderated learning effects on word reading fluency, their statistical learning moderated word reading fluency, decoding and spelling, while their bilingual group interacted with the intervention for learning of word reading accuracy, fluency and decoding. The particular moderating effects did not specifically align with our hypotheses.

### Phonological Awareness

When children’s phonological awareness level was taken into account, word reading fluency outcomes were moderated by this skill, which has been reported to be foundational to learning to read in alphabetic languages. We hypothesized that phonological awareness may be more relevant for learning with the phoneme-level intervention, since lexical strategies could be used for the word-level intervention activities. As such, phonological awareness was expected to positively moderate outcomes for the phoneme-level group. However, for word reading fluency, children with higher phonological awareness scores appeared to benefit more from a word-level focused intervention than one focused at the phoneme-level, whereas those with high phonological awareness benefited less from a rime-level focused intervention. Two possibilities may explain the difference in our results versus previous findings (Olson and Wise, 1992; Kyle et al., 2013). Since the children in this study were simultaneous bilinguals, learning to read in two languages at the onset, the relation between phonological awareness and developing reading skills may be more complex. For example, the reading-phonological awareness relation was found to differ for children learning different sets of languages and scripts (O’Brien et al., 2019). Second, the children in this study may have inherent difficulties accessing the phoneme level of analysis, as do others with reading disorders.

### Bilingual Sets of Language

The other languages that these bilingual, simultaneously biliterate children were learning in school were also expected to exert some influence on how children approach the task of learning to read in English. In this case, we examined the bilingual sets of children, English and Chinese, English and Malay, and English and Tamil learners, in terms of possible moderating effects of these other languages on intervention effects. English–Chinese learners appeared to benefit more from word- versus phoneme-level intervention for word reading, but more from phoneme-level versus word-level intervention for decoding, when compared with English–Malay learners. Word reading fluency also showed longer term positive outcomes from word-level intervention for the English–Malay learners compared to the English–Chinese learners. In the study noted above, O’Brien et al. (2019) found that early reading skills were predicted by syllable-level phonological awareness for Chinese/English and Malay/English bilingual typically-developing children, whereas phoneme-level awareness was more predictive for Tamil/English bilingual children. The sets of languages that biliterates come to learn have different forms of influence, depending on the linguistic and typological distance and phonological transparency of the scripts (e.g., Yelland et al., 1993; Bialystok et al., 2005). It is important to tease apart the cross-linguistic and cross-orthographic transfer of skills when considering reading interventions (Bassetti, 2013). Rickard Liow and Lau (2006) note that conventions for teaching reading, based on monolingual research on alphabetic systems, may not be as useful for bilingual children, and that other forms of metalinguistic awareness, such as morphological awareness, may be more important in some cases. Research in this area is only beginning, but will inform more viable interventions for a variety of learners.

### Statistical Learning

The other main learner characteristic that we considered was statistical learning. We hypothesized that statistical learning may be most beneficial when learning with the rime-level intervention, because picking up orthographic patterns should be easier for those with greater statistical learning ability. When children’s level of statistical learning was taken into consideration, three outcome measures showed differential treatment effects over time. These included decoding, spelling and word reading fluency. Those with lower statistical learning skill benefited more from the phoneme-level intervention in each case. On the other hand, those with higher statistical learning showed better longer term outcomes from a rime-level intervention at follow-up. The effects that statistical learning had across several outcome measures suggest that this is an important skill which may moderate learning and intervention. However, the construct of statistical learning is still unclear from the literature, and an understanding of its contribution to reading acquisition is still developing (see Siegelman et al., 2017). Nonetheless, further study is warranted.

### Technology Derived Data

What is unique about the process of using technology-based methods for instruction of reading is that it offers flexible ways to present text, outside of the stationary blots of ink on paper with traditional methods. Fonts with increased interletter spacing have been promoted to ease eligibility for dyslexic readers (Zorzi et al., 2012); reverse contrast enables faster reading for low vision readers (Legge, 2007); synthesized speech helps multiple groups to go beyond what is undecodable text (Meyer and Bouck, 2014) thus allowing customization of the presentation of print.

In addition, technology-based intervention allows educators to see how individual children are performing longitudinally on a trial-by-trial, and session-by-session level while completing the learning activities. This gives a finer-grained assessment of what children can do or continue to struggle with. For example, we observed the types of letter confusions children tended to make when performing the phoneme- and rime-level activities, and how these intervention groups compared on mini-assessments at multiple grain sizes. These types of measures could offer a clearer microgenetic analysis of learning throughout the intervention, as opposed to aggregated two-point pre-test and post-test measures, which may be less sensitive.

Overall, technology-based approaches can be instrumental as a bridge between the laboratory and the classroom. The way we use technology applications can help us to test the hypotheses that we would normally run in the lab, but now have the capacity to do in the classroom with better experimental controls. With a technology approach, using tablets as a medium and gamification as a method, we can bring our research questions to the classroom and actually run real experiments that are ecologically more valid. These experiments can be embedded in an educational setting without disrupting the educational programs of the school. These tools of access, then, allow cognitive scientists to test learning hypotheses in the classroom, with gains for the field and little disruption to the students. These methods may be more easily “scalable” to larger samples as well. Should one approach work, it could be adapted to real classrooms with direct benefits for education, and not only to basic science.

### Limitations

The conclusions we draw from this study need to be considered in light of some limitations. First, our bilingual group of English and Tamil learners was small, so that our comparisons across the language groups was limited to comparing biscriptal bilinguals learning different types of orthographies (Chinese and English) with monoscriptal bilinguals learning two alphabetic orthographies and one script (Malay and English). Research on literacy development for Akshara scripts is limited, so future studies including biliterates acquiring this type of script are needed. Also, the measure we used for statistical learning differences was based on the standard task used in previous literature (Arciuli and Simpson, 2011), but this measure has recently come under more scrutiny (Siegelman et al., 2017). We scored the task in a manner that we expect would highlight individual differences, but future studies should consider alternate ways of measuring such individual differences and perhaps with multiple tasks, if statistical learning is indeed a multi-faceted construct. Finally, the use of technology-based programs has the distinct benefit of individualizing intervention to students’ needs, but comparison of outcomes across children may be affected by differential exposure to different levels of the games. That is, the games were adapted to individual performance, and children could only move on to the next level when they achieved mastery (80% accuracy).

## Conclusion

There are concerns with the advent of widespread use of technology for reading. Just as Socrates was concerned that literacy would lead to the demise of memory skills, current concerns are expressed for how the nature of reading will change with digital text (e.g., Wolf, 2018). Deep reading, that is the feeling of being immersed in a novel or reading for deep understanding of a topic, is at stake. Because reading yields many benefits for the mind, from vocabulary to verbal skills to declarative knowledge (Cunningham and Stanovich, 2001), the concern is warranted. Therefore, understanding how to encourage lifelong reading habits is a worthwhile pursuit in this digital age.

## Data Availability Statement

The datasets generated for this study are available on request to the corresponding author.

## Ethics Statement

The studies involving human participants were reviewed and approved by NTU-Institutional Review Board at irb@ntu.edu.sg. Written informed consent to participate in this study was provided by the participants’ legal guardian/next of kin.

## Author Contributions

BO’B conceived the original idea, designed the study and analytical approach, performed the analytical calculations, and took the lead in writing the manuscript. MH contributed to the implementation of the experiment, data collection and processing, and wrote the methods and co-designed the figures with BO’B. LO verified the analytical methods and contributed to the interpretation of the results. All authors discussed the results, provided critical feedback, analysis, and contributed to the final manuscript.

## Conflict of Interest

The authors declare that the research was conducted in the absence of any commercial or financial relationships that could be construed as a potential conflict of interest.
