# Supplementary material for: Technology-Based Tools for English Literacy Intervention: Examining Intervention Grain Size and Individual Differences
Source: Front Psychol. 2019 Nov 26;10:2625. doi: 10.3389/fpsyg.2019.02625 (PMC6889115; doi:10.3389/fpsyg.2019.02625)

**Appendix**

*Training Phase for Statistical Learning task*. Participants viewed a series of briefly presented alien figures (500 ms each with 100 ms intervals between each) and were required to push a button when an alien appeared twice in a row.

500ms


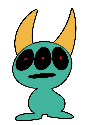


500ms


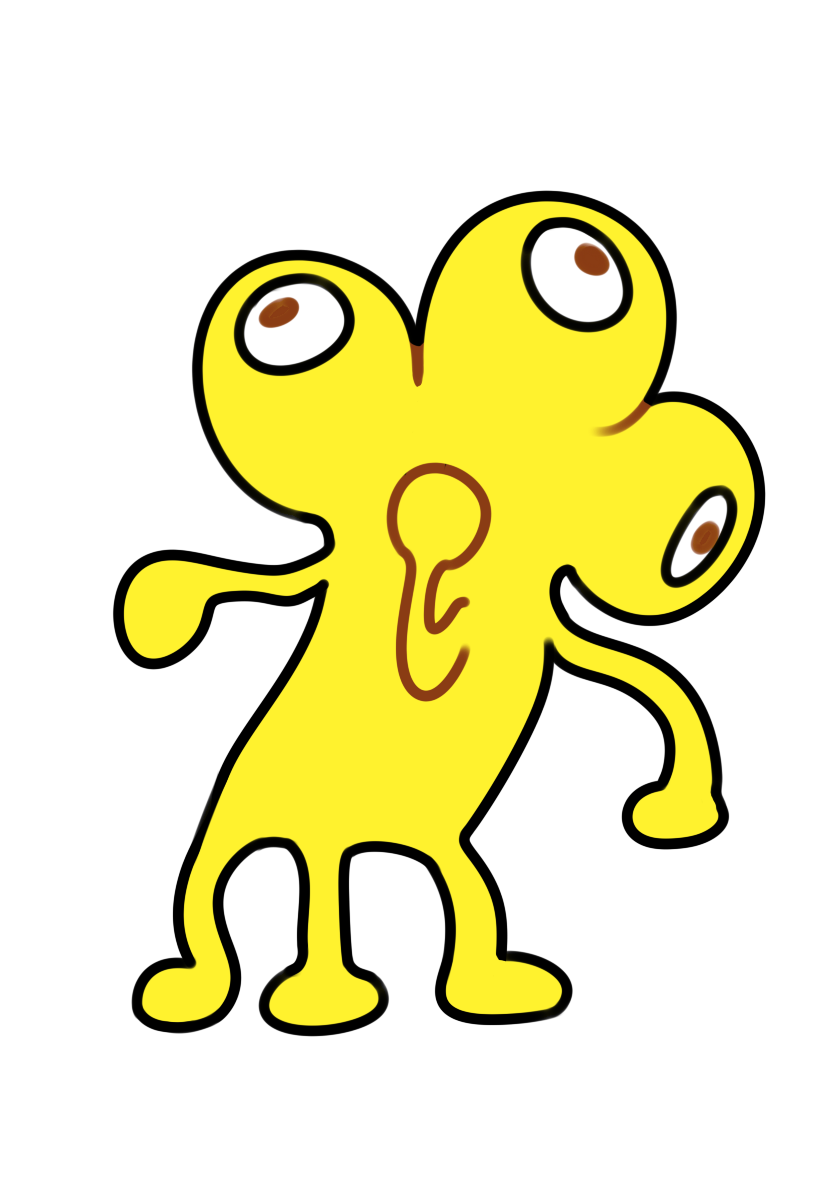


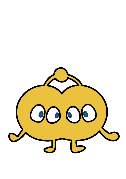


500ms

**…**

500ms


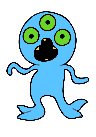


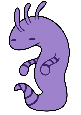


100 ms

500ms

**…**


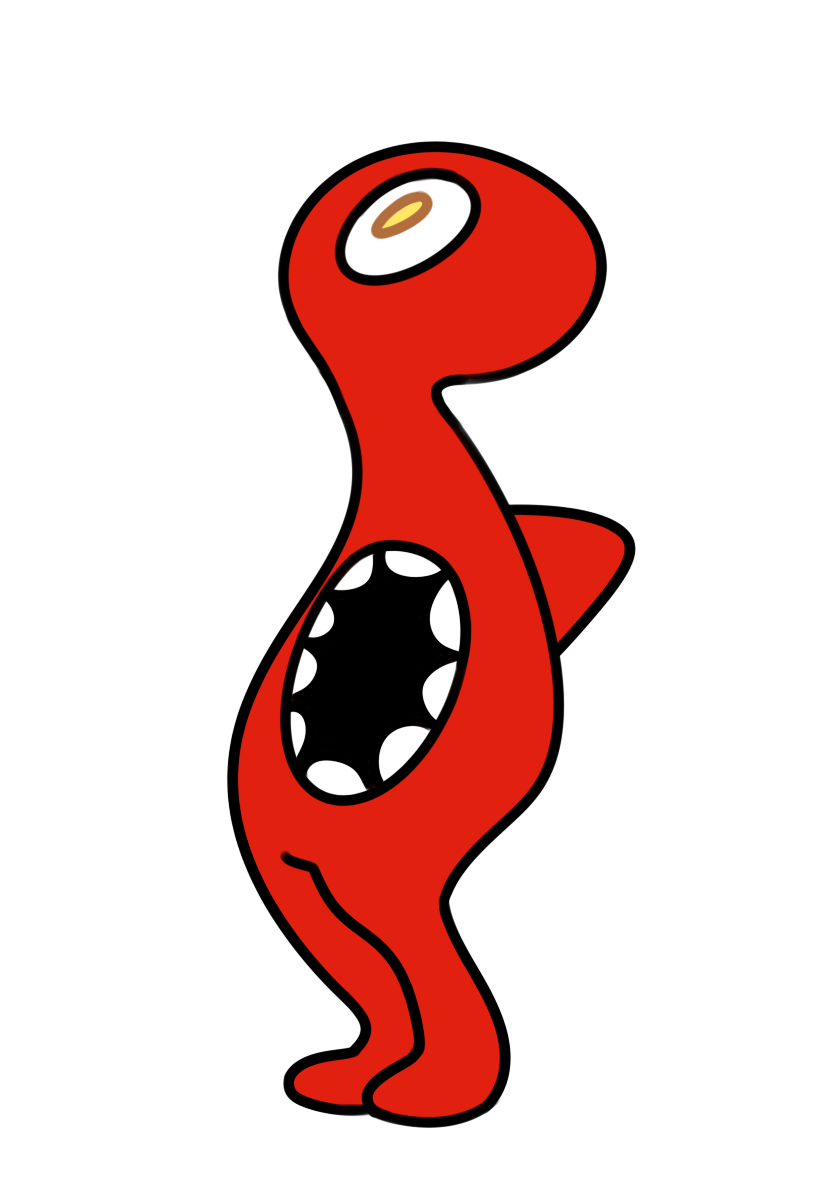


500ms

100 ms


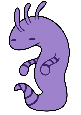
`

500ms

100 ms

**…**

*Test Phase for Statistical Learning task*. After the training phase, participants were asked to choose, which set of aliens had appeared together during the first phase. Triplets were presented in order (1, 2, 3 then 4, 5, 6) with the correct triplet counterbalanced on the left and right side. (The same presentation rate was used as in the training phase: 500 ms per stimulus with 100 ms intervals).


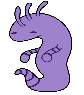


3


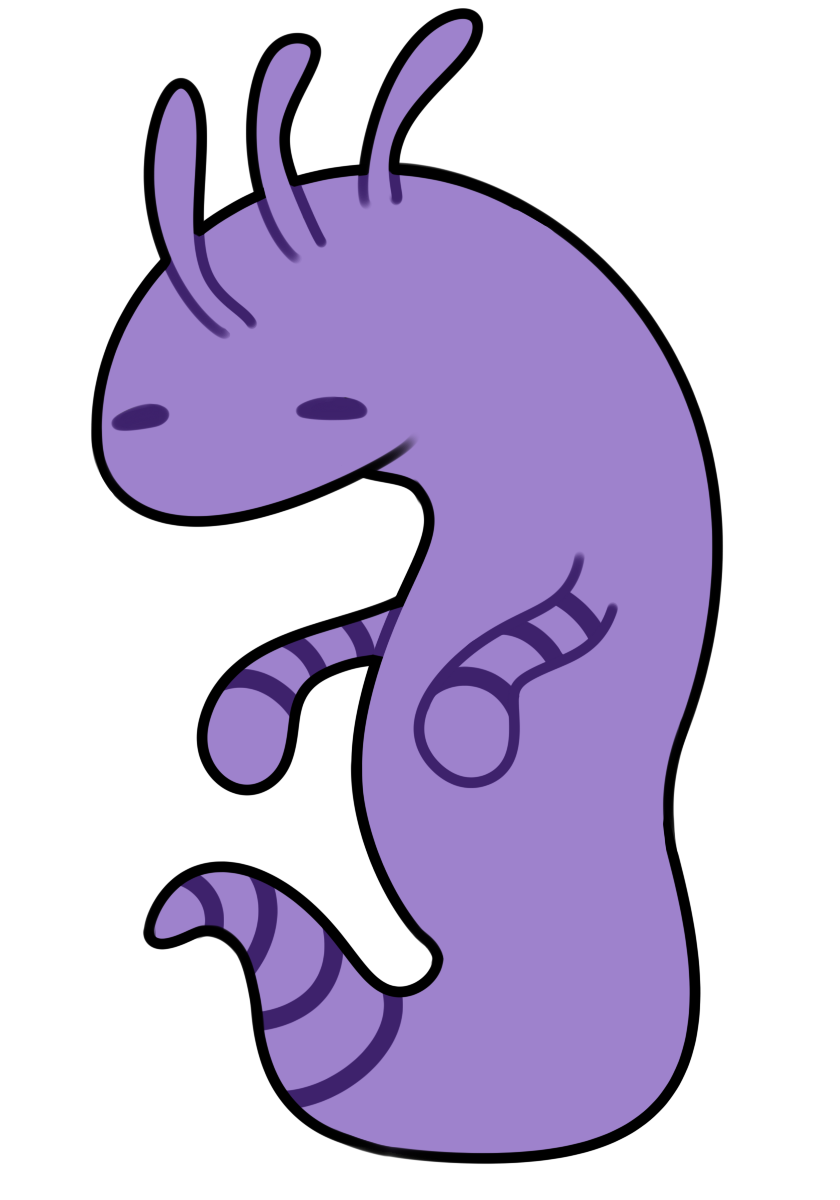


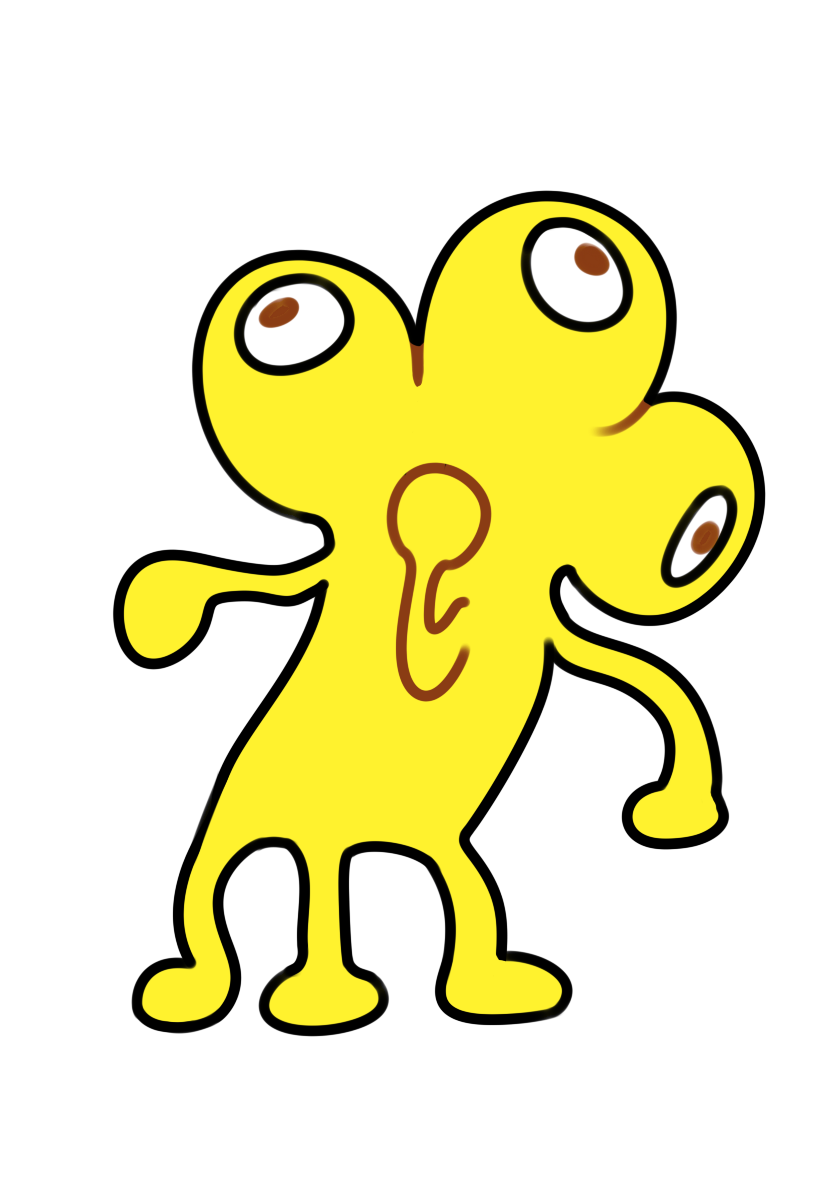


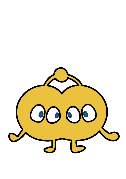

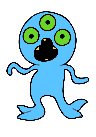


5

6

4

2

1


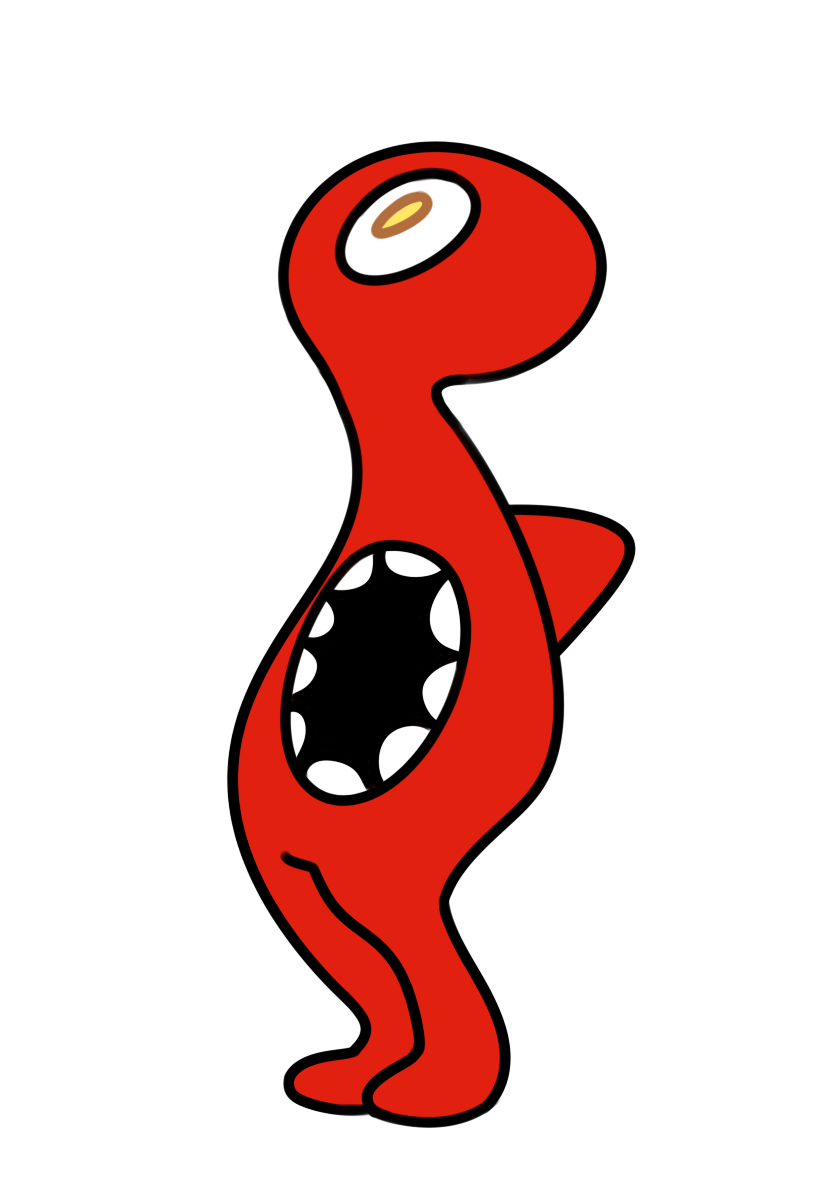


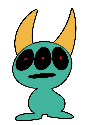


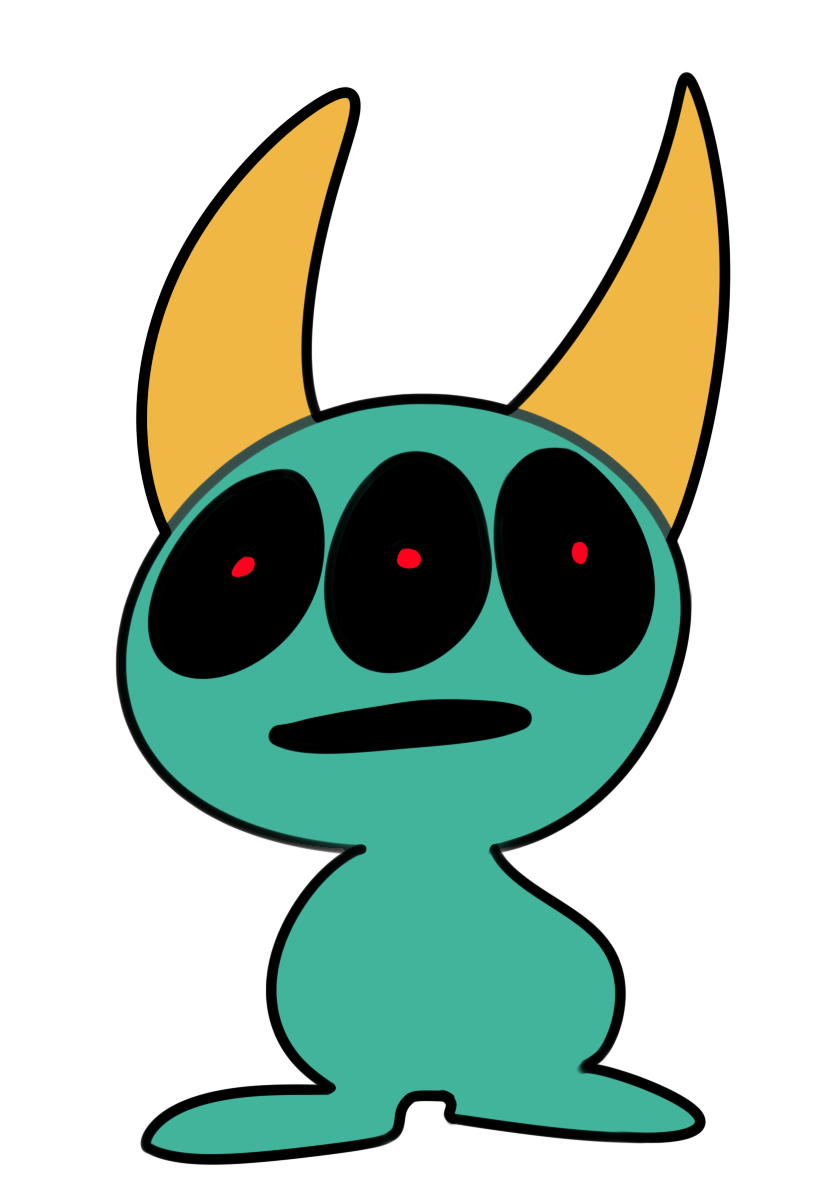

Supplement: Supplementary file 1 [file Data_Sheet_1.docx]
